# Health literacy among main caregivers of Children with Special Health Care Needs: a cross-sectional study *

**DOI:** 10.1590/1518-8345.7860.4689

**Published:** 2025-10-27

**Authors:** Sara Raquel Kuntz, Katarinne Lima Moraes, Alessandra Vaccari, Thailine Stodulski Theis, Ana Luísa Petersen Cogo, Wiliam Wegner

**Affiliations:** 1 Universidade Federal do Rio Grande do Sul, Escola de Enfermagem, Porto Alegre, RS, Brazil.; 2 Hospital de Clínicas de Porto Alegre, Serviço de Enfermagem Pediátrica, Porto Alegre, RS, Brazil.; 3 Universidade de Brasília, Faculdade de Ceilândia, Brasília, DF, Brazil.; 4 Scholarship holder at the Conselho Nacional de Desenvolvimento Científico e Tecnológico (CNPq), Brazil.

**Keywords:** Health Literacy, Child, Chronic Disease, Caregivers, Pediatric Nursing, Patient Safety

## Abstract

to explore health literacy among main caregivers of Children with Special Health Care Needs by identifying limitations and potentialities and their relationship with socioeconomic variables, information sources and social support.

a cross-sectional, descriptive and analytical study conducted with 139 caregivers. The data were collected between December 2022 and April 2023 in a hospital from southern Brazil, by means of the Children with Special Health Care Needs Screener, the Health Literacy Questionnaire and a characterization questionnaire. The analysis involved descriptive and inferential statistics.

a number of limitations were identified in active care, social support and ability to find good health information. Diverse potentialities were noticed in assessing the information and interacting with health professionals. There was a positive association between the “Schooling level” and “Family income” variables and the “Feel understood and supported by healthcare providers” ( *p* =0.006), “Have social support for health” ( *p* =0.000) and “Appraise health information” ( *p* =0.038) scales.

the interventions for caregivers should strengthen active care, social support and access to information, considering the influence exerted by the socioeconomic factors and the importance of good communication with health professionals to improve access, understanding and leverage of the guidelines, thus enhancing care quality and safety.

## Highlights

**(1)** Schooling level and family income exert an influence on health literacy conditions. **(2)** Internet was found to be the caregivers’ main health information source. **(3)** The caregivers’ ability to find good health information on their own is limited. **(4)** Limited social aid and support is provided to CSHCN caregivers.

## Introduction

Health literacy is recognized as an important and modifiable social determinant of health, establishing a relationship with quality of life in those cared for. It is understood as the knowledge and personal skills acquired through everyday activities, social interactions and generations, which enable people to access, understand, assess and use diverse information and services, so that well-being and good health are maintained and promoted, both for themselves and for those around them. Knowledge and personal skills are mediated by organizational structures and by resource availability ^( 1 )^ .

A number of studies point to the need to acknowledge health literacy as something that transcends each person. It involves understanding that the personal skills required to deal with health issues are influenced by the environment in which they are applied. Therefore, the interaction between the health system’s complexity/demands and people’s skills should be considered ^( 2 - 3 )^ .

Inadequate health literacy is considered a public health problem. Consequently, it is relevant to acknowledge the population’s health literacy conditions, so that education and planning in public health can be improved ^( 2 )^ . Caregivers of children with complex chronic diseases represent a critical group in the population because, in addition to being responsible for their own life and health, they should also zeal for the health and well-being of the children under their care ^( 4 - 5 )^ .

The Maternal and Child Health Bureau (MCHB) from the United States of America (USA) designated this population segment as Children with Special Health Care Needs (CSHCN), *Crianças com Necessidades Especiais de Saúde* (CRIANES) ^( 6 )^ in Brazil. Currently, the term “Children with Special Health Care Needs” (adopted in this study) has been employed based on an epistemological movement that understands needs as inherent to health status and not to children themselves, as indicated in contemporary studies ^( 7 - 8 )^ . These children are at a high risk of presenting chronic, physical, developmental, behavioral or emotional conditions or already suffer from them, require special and permanent care to maintain their health and need to use health services and see different professionals from multiple specialties more, beyond what children habitually demand ^( 6 , 9 )^ .

Given these conditions, such children strongly depend on their caregivers’ skills in terms of understanding and critically assessing health information, of their communication with health professionals and of how to navigate the health system ^( 10 )^ . Thus, in order to assess health literacy conditions among CSHCN caregivers, it is indispensable to employ an encompassing instrument that incorporates the multidimensionality of health literacy ^( 11 - 12 )^ .

Caregivers of CSHCN face everyday challenges to ensure safe and adequate care, requiring skills to understand and apply diverse health information. However, barriers related to health literacy (such as difficulties gathering and interpreting medical information) can impair decision-making, treatment adherence and children’s safety ^( 4 )^ .

Thus, identifying health literacy conditions in a given population group provides useful and necessary information to health organizations and professionals to improve health actions and services ^( 13 )^ . A number of international studies contribute increasing evidence about the relationship between caregivers’ inadequate health literacy and failure in home-based care management and worse health results among these children, related to low treatment adherence, errors in drug administration at home and more unscheduled hospitalizations ^( 14 - 17 )^ . Nonetheless, this situation is little known in Brazil and there is scarcity of studies showing the health literacy conditions among this population group, thus justifying the need to conduct this study.

This research seeks to fill this gap by identifying the caregivers’ limitations and potentialities in navigating health systems, gathering and applying diverse information and making sound decisions for the care of these children. Therefore, the study objective was to explore health literacy among caregivers of Children with Special Health Care Needs by identifying limitations and potentialities and their relationship with socioeconomic variables, information sources and social support.

## Method

### Study design

This is a quantitative, cross-sectional, descriptive and analytical study ^( 18 )^ , reported according to the guidelines set forth in Strengthening the Reporting of Observational Studies in Epidemiology (STROBE) ^( 19 )^ .

### *Locus* and period 

The study was conducted between December 2022 and April 2023 in Porto Alegre, RS (Brazil). It was developed in the pediatric units from a high-complexity hospital. The Pediatrics service has 131 beds and is a nationwide reference for the care of children with medium- and high-complexity chronic diseases such as gastrointestinal, genetic, oncological, hematological, neurological and liver ones, as well as candidates for liver transplant and transplanted patients or for hematopoietic stem-cell transplant, among other chronic diseases.

### Population

Main caregivers of Children with Special Health Care Needs, hospitalized in the aforementioned pediatric units.

### Criteria to select and define the sample

By “main caregiver” we understand any single person that identifies themselves as responsible for a child and for providing everyday care in a permanent or partial way, unpaid and not necessarily with blood ties, but emotional. Individuals aged less than 12 years old during the data collection period were considered children ^( 20 )^ . In order to determine their eligibility as CSHCNs, it was defined that they should be included in at least one of the domains indicated by applying the Brazilian version of the Children with Special Health Care Needs Screener (CSHCN *Screener* ®) instrument among all three existing ones, namely: medication use, attending health services and functional limitations ^( 21 )^ .

The electronic medical records of the patients hospitalized in the pediatric units were first consulted to identify those aged less than 12 years old. Subsequently, the Brazilian version of the CSHCN Screener instrument ^®(21)^ was applied to screen CSHCNs. At that same moment, the caregiver characterization instruments and the health literacy assessment questionnaire (HLQ-Br) were applied to the main caregivers of the children identified as CSHCNs.

The subjects included in the study were those aged over 18 years old, having Brazilian Portuguese (language of the instruments’ validated versions) as their first usual tongue and identifying themselves as main caregivers (either during hospitalization or at home) of CSHCNs aged less than 12 years old that had already been discharged from the hospital to their homes at least once. No exclusion criteria were established to select the sample.

The minimum number of participants calculated for the sample was 124 caregivers, considering a 10% increase for possible losses and refusals, thus totaling 138 participants. 80% power, 5% significance level and Cohen’s equal to 0.51 were considered in the calculation ^( 22 )^ . This calculation was performed in the PSS Health tool, online version ^( 23 )^ .

### Data collection instruments and study variables

The Brazilian version of the CSHCN Screener® instrument was applied to identify hospitalized CSHCNs. This is a screening instrument originally developed in English and translated/adapted to Brazilian Portuguese, which showed good internal consistency. It manages to identify and assess the care demands of Children with Special Health Care Needs in relation to a given chronic condition, regardless of any clinical diagnosis ^( 6 , 21 )^ . Choice of this instrument was warranted given its sensitivity in identifying the multiple dimensions inherent to children’s health needs. This includes the increased risk for chronic conditions criterion, according to the original definition of CSHCN, which considers conditions lasting at least 12 months. Although the children included in the sample already suffered from installed chronic conditions, the instrument was applied to confirm their eligibility at the collection moment and identify the “Special health care needs” domains, as it was a study aimed at assessing health literacy among caregivers of children already identified as with special health care needs.

CSHCN Screener® includes five items with two subitems each (a and b), except item five, which has only one. All the answer options are of the “Yes” or “No” type. The subitems are formulated through affirmative answers in each corresponding item and seek relationships between these assertions and the currency of any health problem with actual or expected duration of at least twelve months. If an affirmative answer is given to any of the items (along with its corresponding subitems), this already indicates that the child has some special health care need. The five items are distributed across three “Special health care needs” domains that demand specialized assistance in terms of health and educational services, namely: medication use, attending health services and functional limitation ^( 21 )^ .

A CSHCN and caregiver characterization questionnaire specially developed for this study was applied, considering the relevance of knowing each family’s socioeconomic, demographic and cultural factors and the children’s health history for an accurate diagnosis regarding the caregivers’ health literacy. The questionnaire includes variables on the following aspects: CSHCN characterization [origin of the special health care needs (congenital/acquired cause and medical diagnosis), categories of the care demands (developmental, technological, medication-related, modified, mixed and clinically-complex habitual demands), reason for the current hospitalization, hospitalization and birth date, gender, rehospitalization reason and date after first hospital discharge, number of emergency visits or hospitalizations in the last year and their reasons, use of other health services]; and caregiver socioeconomic characterization [kinship with the CSHCNs, birth date, gender, schooling, profession/occupation, family income, having other children, other people with special health care needs depending on them, main health information sources (TV, journal, radio, Internet and others), residence city and state and presence of another main caregiver].

The Brazilian version of the Health Literacy Questionnaire (HLQ-Br) was applied to assess the main caregivers’ health literacy conditions. The Health Literacy Questionnaire is a multidimensional instrument that has been translated to Brazilian Portuguese and validated with 794 adult users of the Unified Health System ( *Sistema Único de Saúde* , SUS) from three Brazilian regions. Good psychometric properties were obtained in the validation process. It is a multidimensional instrument, with 44 items distributed across nine scales that are subdivided into two parts. Part 1 has four answer options, from “I totally disagree” to “I totally agree” (scores 1-4); and Part 2 offers five answer options, from “I can’t do it or I always find it difficult” to “I always find it easy” (scores 1-5). This instrument does not provide a global score but values with the individual mean of the nine scales. Scores closer to the upper or lower limits indicate the caregivers’ potentialities and limitations in self-healthcare, respectively ^( 24 )^ .

Due authorization from the University of Deakin (Australia) was obtained to apply the Brazilian version of HLQ, via the “ HLQ-info@swin.edu.au ” email address.

### Data collection

The data were collected from the patients’ electronic medical records and by means of interviews with the main caregivers of CSHCNs, conducted in a private room at the pediatric units. The research team was previously trained to collect the data from the medical records and to apply the questionnaires.

### Data treatment and analysis

The data were analyzed in the Statistical Package for the Social Sciences (SPSS/PASW), version 27.0. Mean and standard deviation values were used in the descriptive analysis of the quantitative variables; in turn, absolute and relative frequencies were employed to analyze the categorical variables. Choice of the distribution type was based on the Standard Deviation size (not exceeding half of the mean value to consider symmetric distribution) and on proximity between mean and median values (difference of less than 20%). A 5% significance level (p≤0.05) was adopted.

The Student’s t test or Analysis of Variance (ANOVA) were applied to compare mean values, along with Tukey’s test. In addition, Pearson’s chi-square or Fisher’s exact tests were applied to evaluate the association between the categorical variables. In turn, Spearman’s correlation test was employed to assess the association between the ordinal variables and the HLQ-Br scores.

### Ethical aspects

This study had its project approved by the Research Ethics Committee of the institution researched, under Opinion No. 5,751,478. All ethical aspects were respected and followed according to the current legislation. The participants signed a Free and Informed Consent Form.

## Results

The sample included 139 main caregivers of hospitalized CSHCNs. Most of these caregivers were female (89.9%) and aged between 31 and 60 years old (62.6%), had Complete High School (55%), were housewives (79%), earned from one to two minimum wages as family income (73.4%) and lived in different municipalities from the Brazilian South region (95.6%).

In the sample, 39 caregivers (28.1%) had some other person with special health care needs under their care, in addition to the CSHCN. The main caregivers were predominantly mothers (84.2%) with other children in addition to the CSHCN (69.1%), and 86 main caregivers (61.9%) reported support from a second main caregiver for the assistance to be provided to the child: the father in 38.1% of the cases.

The participants were main caregivers of Children with Special Health Care Needs related to medication use due to some chronic condition (94.2%), to using health services more than most of the children of the same age (100%) and to having some functional limitation or inability to perform activities common to most children (53.2%). In all, 137 (97.8%) of these children had more than one special health care need and 98 (70.5%) were also technology-dependent in use of at least one medical device.

In the group analysis of the “Special health care needs” domains corresponding to the CSHCNs, only three (2.2%) needed Health Services (HSs) alone; five (3.6%) required Health Services (HSs) and had some Functional Limitation (FL); 62 (44.6%) resorted to Medication Use and HSs and most of the CSHCNs (69; 49.6%) had some FL and required Medication Use and HSs.

As described in Table 1 and considering the mean scores obtained in all nine scales from the HLQ-Br questionnaire, limitations in the caregivers’ health literacy were identified in relation to Actively managing health (Scale 3 - Part 1), have social support for health (Scale 4 - Part 1) and Ability to find good health information (Scale 8 - Part 2). Potentialities in the caregivers’ health literacy were detected in terms of Appraise health information (Scale 5 - Part 1) and Ability to actively engage with healthcare providers (Scale 6 - Part 2).

**Table 1-:** Mean scores obtained in the HLQ-Br* scales and items indicating greater health literacy limitations and potentialities among the main caregivers of hospitalized CSHCNs ^†^ (n = 139). Porto Alegre, RS, Brazil, 2023

**HLQ-Br** * **scales and questions**	**Mean**	**Standard Deviation**
**Part 1 (Scores 1-4)**		
**Scale 1 – Feel understood and supported by healthcare providers**	3.02	0.53
Q ^‡^ 17 – I have the health professionals I need to…	3.25	0.71
Q ^‡^ 8 – I have at least one health professional with whom to…	2.79	0.91
**Scale 2 – Have sufficient information to manage my health**	3.03	0.47
Q ^‡^ 23 – I have all the information I need to…	3.68	0.64
Q ^‡^ 14 – I am certain that I have all the information that…	3.01	0.73
**Scale 3 – Actively managing health**	2.82	0.51
Q ^‡^ 6 – I spend much time with…	3.40	0.95
Q ^‡^ 21 - I do something regularly to…	2.51	0.70
**Scale 4 – have social support for health**	2.96	0.59
Q ^‡^ 19 – I have strong support…	3.24	0.76
Q ^‡^ 5 – When I feel ill, people around me…	2.75	0.78
**Scale 5 – Appraise health information**	3.13	0.45
Q ^‡^ 7 – When I see new health information…	3.37	0.62
Q ^‡^ 4 – I compare all the health information I get…	2.94	0.76
**Part 2 (Scores 1-5)**		
**Scale 6 – Ability to actively engage with healthcare providers**	4.00	0.57
Q ^‡^ 20 – Asking questions to health professionals to…	4.39	0.83
Q ^‡^ 2 – Being certain that health professionals…	3.60	0.96
**Scale 7 – Ability to navigate the healthcare system**	3.70	0.74
Q ^‡^ 13 – Being certain to find the right place to…	4.01	1.07
Q ^‡^ 1 – Finding the health service…	3.31	1.08
**Scale 8 – Ability to find good health information**	3.60	0.64
Q ^‡^ 10 – Finding health information to…	3.81	0.91
Q ^‡^ 18 – Finding information about…	3.37	1.19
**Scale 9 – Ability to understand health information well enough to know what to do**	3.99	0.57
Q ^‡^ 21 – Understanding that health professionals are…	4.38	0.71
Q ^‡^ 12 – Reading and understanding information…	3.81	1.06

*HLQ-Br = Brazilian version of the Health Literacy Questionnaire; ^†^ CSHCNs = Children with Special Health Care Needs; ^‡^ Q = Question

All the participating caregivers had gone through at least one hospitalization with the CSHCNs in the last 12 months prior to data collection in this study. Of them, 30 (21.6%) were hospitalized only once, 74 (53.2%) from two to six times, 23 (16.5%) from seven to eleven times and 12 (8.7%) had to be hospitalized at least twelve times during that period.

Table 2 describes the correlation coefficients between the mean scores obtained in the nine HLQ-Br scales and the mean variables corresponding to the number of hospitalizations of the CSHCNs and to the main caregivers’ schooling levels and family incomes. The positive and statistically significant associations (p<0.05) indicate the relationship of the caregivers’ schooling level and family income conditions with their health literacy limitations and potentialities. They are also indicative in relation to the number of hospitalizations required for each CSHCN.

**Table 2-:** Correlation of the mean scores obtained in the HLQ-Br* scales with the socioeconomic characteristics of the CSHCN ^†^ caregivers and the number of times these children were hospitalized (n = 139). Porto Alegre, RS, Brazil, 2023

HLQ-Br* scales	**Schooling level**	**Family income**	**Hospitalizations of the CSHCNs** ^†^
**Part 1**			
**1 -** Feel understood and supported by healthcare providers	r _s_^‡^ =0.23 ^§^	r _s_^‡^ =0.23 ^§^	r _s_^‡^ =0.16
	(p=0.006)	(p=0.006)	(p=0.057)
**2 -** Have sufficient information to manage my health	r _s_^‡^ =0.15	r _s_^‡^ =0.09	r _s_^‡^ =0.17
	(p=0.084)	(p=0.302)	(p=0.053)
**3 -** Actively managing health	r _s_^‡^ =0.10	r _s_^‡^ =0.09	r _s_^‡^ =0.08
	(p=0.260)	(p=0.292)	(p=0.334)
**4 -** Have social support for health	r _s_^‡^ =0.33 ^§^	r _s_^‡^ =0.32 ^§^	r _s_^‡^ =-0.004
	(p=0.000)	(p=0.000)	(p=0.96)
**5 -** Appraise health information	r _s_^‡^ =0.17 ^||^	r _s_^‡^ =0.17 ^||^	r _s_^‡^ =0.19 ^||^
	(p=0.038)	(p=0.044)	(p=0.019)
**Part 2**			
**6 -** Ability to actively engage with healthcare providers	r _s_^‡^ =0.13	r _s_^‡^ =0.15	r _s_^‡^ =-0.12
	(p=0.115)	(p=0.064)	(p=0.158)
**7 -** Ability to navigate the healthcare system	r _s_^‡^ =0.62	r _s_^‡^ =0.20 ^||^	r _s_^‡^ =-0.051
	(p=0.467)	(p=0.016)	(p=0.553)
**8 -** Ability to find good health information	r _s_^‡^ =0.35 ^§^	r _s_^‡^ =0.26 ^§^	r _s_^‡^ =-0.01
	(p=0.000)	(p=0.002)	(p=0.837)
**9 -** Ability to understand health information well enough to know what to do	r _s_^‡^ =0.22 ^§^	r _s_^‡^ =0.16 ^||^	r _s_^‡^ =0.01
	(p=0.009)	(p=0.049)	(p=0.838)

*HLQ-Br = Brazilian version of the Health Literacy Questionnaire; ^†^ CSHCNs = Children with Special Health Care Needs; ^‡^ r _s_ = Spearman’s correlation coefficient; ^§^ Significant correlation at the 0.01 level; ^||^ Significant correlation at the 0.05 level

In relation to Part 1 of the HLQ-Br questionnaire, there was a positive and statistically significant difference between the “Schooling level” and “Family income” variables and the following scales: 1 - Feel understood and supported by healthcare providers (p=0.006), 4 - Have social support for health (p=0.000) and 5 - Appraise health information (p=0.038; p=0.044). There was also a positive and significant association between the “Hospitalizations of the CSHCNs” variable and Scale 5 – Appraise health information (p=0.019) ( Table 2 ).

As for Part 2, a positive and statistically significant association was identified between the “Schooling level” and “Family income” variables and the following scales: 8 - Ability to find good health information (p=0.000; p=0.002) and 9 - Ability to understand health information well enough to know what to do (p=0.009; p=0.049). An association was also found between the “Family income” variable and Scale 7 - Ability to navigate the healthcare system (p=0.016) ( Table 2 ).

Most of the caregivers stated using Internet (87%) as one of their main health information sources; in turn, health professionals were mentioned as the main source by 19 caregivers (13.6%) and three (2.1%) indicated other main caregivers as one of their main health information sources.

Potentialities in health literacy in terms of “Feel understood and supported by healthcare providers” (Scale 1) were identified in those caregivers that stated resorting to these professionals as one of their main health information sources (p=0.002). In turn, as for “Appraise health information” (Scale 5), potentialities in health literacy were observed among those caregivers that mentioned Internet (p=0.026) as one of their main health information sources ( Table 3 ).

Limitations in health literacy in terms of “Have social support for health” (Scale 4)” and of “Appraise health information” (Scale 5) were identified in those caregivers that stated not having support from a second main caregiver for the assistance to be provided to CSHCNs (p=0.039; p=0.037), according to the means comparisons described in Table 3.

**Table 3-:** Association of the health literacy conditions among CSHCN* caregivers according to the HLQ-Br ^†^ questionnaire and the “Support network for health” and “Health information sources” variables (n = 139). Porto Alegre, RS, Brazil, 2023

**HLQ-Br** ^†^ **scales**	**S** ^‡^ **1**	**S** ^‡^ **2**	**S** ^‡^ **3**	**S** ^‡^ **4**	**S** ^‡^ **5**	**S** ^‡^ **6**	**S** ^‡^ **7**	**S** ^‡^ **8**	**S** ^‡^ **9**
**Variables**	**M** ^§^ ±SD ^||^	**M** ^§^ ±SD ^||^	**M** ^§^ ±SD ^||^	**M** ^§^ ±SD ^||^	**M** ^§^ ±SD ^||^	**M** ^§^ ±SD ^||^	**M** ^§^ ±SD ^||^	**M** ^§^ ±SD ^||^	**M** ^§^ ±SD ^||^
**HIS** ^¶^ - **Another caregiver**									
No	2.97±0.51	3.01±0.52	2.73±0.53	2.83±0.58	3.03±0.43	4.04±0.54	3.74±0.76	3.58±0.59	4.04±0.50
Yes	3.06±0.55	3.04±0.44	2.88±0.49	3.05±0.59	3.10±0.45	3.97±0.59	3.68±0.73	3.60±0.67	3.97±0.60
p-value**	0.366	0.750	0.086	0.039	0.037	0.448	0.618	0.855	0.489
**HIS** ^¶^ **- Internet**									
No	2.87±0.44	3.06±0.49	2.93±0.49	2.97±0.56	2.91±0.51	4.00±0.61	3.96±0.93	3.55±0.74	4.10±0.54
Yes	3.05±0.54	3.02±0.47	2.81±0.51	2.96±0.59	3.16±0.43	4.00±0.57	3.66±0.70	3.60±0.62	3.98±0.57
p-value**	0.192	0.711	0.349	0.925	0.026	0.99	0.115	0.74	0.42
**HIS** ^¶^ **- Journal/Book**									
No	3.02±0.52	3.02±0.46	2.83±0.50	2.97±0.58	3.10±0.44	4.00±0.58	3.71±0.74	3.57±0.64	3.99±0.56
Yes	3.04±0.62	3.04±0.56	2.78±0.67	2.83±0.62	3.40±0.47	4.00±0.56	3.63±0.77	3.90±0.47	4.03±0.66
p-value**	0.915	0.914	0.760	0.452	0.041	0.993	0.751	0.098	0.820
**HIS** ^¶^ **- TV/Radio**									
No	3.06±0.53	3.04±0.46	2.83±0.49	2.97±0.59	2.94±0.59	3.15±0.42	3.74±0.69	3.60±0.61	4.03±0.50
Yes	2.91±0.523	2.98±0.49	2.80±0.56	2.94±0.59	3.07±0.52	3.80±0.56	3.60±0.85	3.59±0.72	3.88±0.71
p-value**	0.14	0.511	0.707	0.815	0.333	0.013	0.335	0.940	0.165
**HIS** ^¶^ **- Health professional**									
No	2.97±0.52	3.01±0.49	2.85±0.50	2.98±0.58	3.11±0.45	4.00±0.58	3.72±0.75	3.61±0.65	4.01±0.58
Yes	3.38±0.49	3.13±0.30	2.66±0.52	2.87±0.62	3.24±0.46	3.95±0.55	3.57±0.63	3.50±0.55	3.90±0.48
p-value**	**0.002**	0.320	0.134	0.468	0.458	0.725	0.397	0.484	0.446
**HIS** ^¶^ **- Caregivers**									
No	3.02±0.53	3.03±0.47	2.84±0.50	2.97±0.59	3.12±0.45	4.00±0.58	3.71±0.74	3.60±0.64	3.99±0.57
Yes	3.25±0.47	2.91±0.62	2.13±0.46	2.60±0.34	3.40±0.52	4.06±0.41	3.22±0.63	3.53±0.46	4.00±0.52
p-value**	0.47	0.676	0.017	0.280	0.304	0.844	0.257	0.853	0.997

*CSHCNs = Children with Special Health Care Needs; ^†^ HLQ-Br = Brazilian version of the Health Literacy Questionnaire; ^‡^ S = Scale; ^§^ M = Mean; ^||^ SD = Standard Deviation; ^¶^ HIS = Health Information Source; **p-value = Student’s t test

## Discussion

The study innovation lies in having analyzed health literacy among CSHCN caregivers by resorting to a multidimensional assessment instrument. Therefore, it was possible to recognize the limitations and potentialities in terms of these caregivers’ functional, interactive and critical skills, associating them with their socioeconomic levels and with the care demands that are necessary for CSHCNs ^( 2 )^ .

CSHCNs require high demand of specific and complex care measures; in addition to that, they need multiprofessional treatments and monitoring for extended periods of time. Leading this complex scenario requires specific knowledge and technical/communication skills from the main caregivers ^( 25 - 26 )^ .

All the CSHCNs in this study were identified as dependent on health services and present a significant number of hospitalization during a one-year period. Most of the CSHCNs were recognized when combining the three “Special health care needs” domains, in addition to their dependence on life-maintaining technological devices. Such findings evidence the care complexity required for these children and which are frequently demanded from home caregivers, without due preparedness for dehospitalization or adequate support from health service networks ^( 27 - 29 )^ .

Main caregivers are acknowledged as a fundamental component to provide safe and good quality home-based care to CSHCNs. Along with health professionals, they play a crucial role in decision-making regarding these children’s health, especially in managing and meeting home-based care demands. In addition to the challenges resulting from care complexity, caregivers also face a series of difficulties imposed by the health system itself ^( 15 , 30 - 31 )^ .

In order to meet these demands and overcome the difficulties imposed, caregivers need to develop cognitive and social skills that enable them to navigate the health system, to gather, understand and apply necessary information for care management, and to make judgments and decisions regarding these children’s needs and singularities ^( 26 , 32 )^ .

The results of this study show reasonable health literacy conditions among the caregivers, recognized in the scales from the questionnaire applied, even if limitations related to their functional, interactive and critical skills have also been identified ^( 2 )^ . It is noted that a person’s health literacy is not static. Situational factors such as unknown environments, intimidating interactions and emotional aspects can temporarily and negatively affect these caregivers’ health literacy conditions and ability to manage the necessary care to be provided to CSHCNs ^( 32 - 34 )^ .

As for the interactive and critical skills, there are limitations in relation to “Actively managing health” among the caregivers included in this study. Even if with the necessary skills and knowledge for self-care, these caregivers fail in their actions to promote it, as they do not see it as a priority. Similar findings are identified in other research studies, where main caregivers are exclusively devoted to the intensive complex care demand required by CSHCNs and end up neglecting their own care, which frequently results in physical, emotional and social overload ^( 31 , 35 - 37 )^ .

As expected, the children’s mothers were mainly identified as the main CSHCN caregivers both during hospitalization and at their homes, as already identified in other research studies at the global level ^( 25 - 26 , 34 , 38 )^ . As also detected in other studies ^( 17 , 39 - 41 )^ , the social determinants that presented a significant relationship with these caregivers’ health literacy conditions were schooling level and family income.

Lack of social support also exposes caregivers to physical and mental overload, with the possibility of affecting their health, well-being and quality of life, in addition to hindering the implementation of already acquired health literacy skills and altering their ability to care for CSHCNs ^( 33 , 37 )^ . Most of the caregivers in this study stated enjoying sound support from the primary support network, and even having the assistance of a second caregiver for care relief; however, they also mentioned not feeling understood by their close people when they are ill.

On the other hand, mothers caring for CSHCNs have limited social support and reported not having any support from the children’s fathers for home-based care and feeling significant mental and physical wear out. As for social-family relationships, they state feelings of isolation, lack of understanding/empathy and communication difficulties. Even if without adequate family support, by sharing their experiences these caregivers establish strong ties with other CSHCN mothers that make them feel understood and supported ^( 42 )^ .

Social support from friends and family members was pointed out as essential to manage these children’s needs, share the care provided and, above all, incorporate new information about the challenges when facing the complexity inherent to caring for CSHCNs. More advanced interactive and communication critical skills favor strengthening the primary and secondary support networks and enable deeper information gathering from different sources ^( 32 , 37 )^ .

Weakened secondary social networks also contribute to the caregivers’ health literacy limitations. Some studies identify health and social welfare institutions as the secondary support network and describe the caregiver-health professional relationship as conflicting, weak or broken. The weakness of this relationship is associated with fragmented organization in health services, low-assistance quality, limited responsibility on the part of health professionals, communication gaps, insufficient guidance and distancing from caregivers ^( 37 , 43 )^ .

When speaking about CSHCN caregivers, insufficient skills to find information and navigate the health system on their own constitute an extremely relevant barrier to providing home-based care and indicate limitations in these caregivers’ functional and interactive health literacy ^( 32 )^ . These children need to frequently attend health services and see professionals from multiple specialties. The larger the team of professionals, the more visits to health services that caregivers need to manage, demanding advanced communication and coordination skills ^( 9 , 38 )^ .

The health system itself frequently causes navigation difficulties. Some studies assessing CSHCNs’ therapeutic path identify health professionals’ unpreparedness and the fragmentation, disarticulation, lack of structure and precariousness found in health services as barriers to accessing and navigating health service networks ^( 31 , 44 - 45 )^ . As well as the professional-caregiver interactions, the caregivers perceive communication among the health professionals responsible for the children’s treatments as scarce and insufficient ^( 37 )^ .

In order to meet the care demands required by CSHCNs, caregivers need access to a broad range of specific and oftentimes complex information. Given the communication, preparedness and guidance gaps for transitional care on the part of health professionals and services ^( 46 )^ , the caregivers’ inability to access health information on their own becomes even more worrying.

In this study, most of the caregivers mention Internet as one of their main health information sources and few participants identified health professionals as with this role. The fact that CSHCN caregivers acknowledge digital/social networks and health professionals as information sources was also described in other studies, where the information provided by the professionals is valued, although with guidelines that are scarcely compatible or applicable to the children’s families’ reality. In these cases, they seek to exchange experiences with other caregivers of children with the same or similar needs ^( 47 )^ . As was the case in this study, other CSHCN caregivers were recognized as important alternative sources of information and experience exchanges to qualify the care provided to CSHCNs.

In another study, it was identified that the caregivers of children with chronic diseases are more prone to accessing social networks discussing children’s health, despite classifying them as less reliable online health information sources. Even so, when finding information that contradicts the professionals’ advice, most of the parents reported following the latter or asking them before making any decision ^( 48 )^ .

This research identified potentialities in the caregivers’ health literacy in relation to their ability to actively engage with healthcare providers. Although most of them failed to identify health professionals as health information sources, they feel capable of gathering additional guidelines from them, asking questions to obtain all the information they need. This result is attributed to these caregivers’ extensive experience and contact with health professionals and services, for being frequent users of the same services to meet the care demands of CSHCNs ^( 37 )^ .

Potentialities were also detected in the caregivers’ critical health literacy in terms of their ability to appraise health information, even identifying a statistically significant difference between the scale corresponding to the item from the questionnaire applied and the caregivers that used Internet as a health information source.

It is noted that Internet can be a valuable instrument for communication and to gather health information for these caregivers, consequently developing their social interaction capacity, deepening their health knowledge and improving their autonomy and self-confidence for care management. At the same time, for being an oftentimes insecure and incomplete information source, it can also turn into a significant health risk for caregivers and children alike. Caregivers need to be able to identify good and reliable information sources and to distinguish contradictory data ^( 46 , 49 - 50 )^ . Given this scenario, it becomes relevant to assess digital health literacy among this population group.

This study stands out for conferring voice to CSHCN caregivers, acknowledging their everyday challenges in the search for information and decision-making to ensure their children’s well-being. By using internationally validated instruments, it offers a faithful reflection of these caregivers’ reality, contributing a novel perspective to this issue in Brazil. In addition to its scientific contributions, it reinforces the need to get health professionals closer to families, strengthening these caregivers’ health literacy and autonomy.

The limitations of this study are related to the fact that the data were collected in a single health institution, which may not represent the caregivers’ reality in other contexts (such as those accessing lower complexity services or facing different regional challenges in the health system), in addition to not having implemented a qualitative approach exploring the causes underlying the multidimensional conditions of the caregivers’ health literacy. In addition to that, its cross-sectional design precludes analyzing changes in health literacy.

Therefore, new studies in more health institutions and with mixed and longitudinal approaches are suggested. That would allow developing broad and individualized interventions to meet the CSHCN caregivers’ health literacy needs.

## Conclusion

The study innovation lies in having explored CSHCN caregivers’ multidimensional health literacy nationwide, by using the association of robust and widely used instruments at the international level, both to screen and classify CSHCNs in needs domains and to assess the main caregivers’ health literacy conditions beyond a mere cognitive capability or limitation.

The findings reveal the influence exerted by the caregivers’ schooling levels and family incomes on their health literacy conditions, in addition to identifying Internet as their main information source. In most of the cases, their ability to preserve their own health and well-being is limited and they have little social support, despite having the help of a second caregiver to meet the CSHCNs’ needs. In addition to that, they feel little understood and with no support from close people when it comes to their own health status. Finally, their ability to find good health information on their own is limited.

We should note the relevance of acknowledging the health literacy limitations and potentialities in this population group as an important resource that allows the necessary instrumentalization for health services and professionals to implement assertive educational interventions, as they carry the responsibility of promoting health literacy, strengthening active care, improving access, understanding and leverage of guidelines, and improving care quality and safety.

## Data Availability

Datasets related to this article will be available upon request to the corresponding author.
