# Supplementary material for: Health literacy among main caregivers of Children with Special Health Care Needs: a cross-sectional study
Source: Rev Lat Am Enfermagem. 2025 Oct 27;33:e4689. doi: 10.1590/1518-8345.7860.4689 (PMC12562652; doi:10.1590/1518-8345.7860.4689)
Supplement: Translation

# Translation: Letramento em saúde de cuidadores principais de crianças que necessitam de atenção especial à saúde: estudo transversal*

**DOI:** 10.1590/1518-8345.7860.4690

**Authors:** Sara Raquel Kuntz, Katarinne Lima Moraes, Alessandra Vaccari, Thailine Stodulski Theis, Ana Luísa Petersen Cogo, Wiliam Wegner

**Affiliations:** 1 Universidade Federal do Rio Grande do Sul, Escola de Enfermagem, Porto Alegre, RS, Brasil.; 2 Hospital de Clínicas de Porto Alegre, Serviço de Enfermagem Pediátrica, Porto Alegre, RS, Brasil.; 3 Universidade de Brasília, Faculdade de Ceilândia, Brasília, DF, Brasil.; 4 Bolsista do Conselho Nacional de Desenvolvimento Científico e Tecnológico (CNPq), Brasil.

**Keywords:** Letramento em Saúde, Criança, Doença Crônica, Cuidadores, Enfermagem Pediátrica, Segurança do Paciente

## Abstract

explorar o letramento em saúde dos cuidadores principais de crianças que necessitam de atenção especial à saúde, identificando limitações e potencialidades e relação com variáveis socioeconômicas, fontes de informação e suporte social.

estudo transversal, descritivo e analítico, realizado com 139 cuidadores. Os dados foram coletados entre dezembro/2022 e abril/2023, em um hospital no Sul do Brasil, por meio do *Children with Special Health Care Needs Screener*, do *Health Literacy Questionnaire* e de um questionário de caracterização. A análise envolveu estatística descritiva e inferencial.

identificaram-se limitações no cuidado ativo, suporte social e capacidade de encontrar boas informações sobre saúde. Potencialidades foram observadas na avaliação das informações e na interação com os profissionais de saúde. Houve associação positiva entre as variáveis instrução e renda familiar com as escalas compreensão e apoio profissional de saúde (*p* = 0,006), suporte social para saúde (*p* = 0,000) e avaliação das informações em saúde (*p* = 0,038).

intervenções para cuidadores devem fortalecer o cuidado ativo, suporte social e acesso à informação, considerando a influência dos fatores socioeconômicos e a importância da comunicação com profissionais para melhorar o acesso, compreensão e uso das orientações, aumentando a qualidade e segurança do cuidado.

## Destaques

**(1)** Grau de instrução e a renda familiar influenciam nas condições do letramento em saúde. **(2)** A *internet* como principal fonte de informação sobre saúde dos cuidadores. **(3)** Capacidade limitada de encontrar boas informações sobre saúde por conta própria. **(4)** Disposição de apoio e suporte social limitado aos cuidadores de CRIANES.

## Introdução

O letramento em saúde é reconhecido como um determinante social importante e modificável da saúde, estabelecendo uma relação com a qualidade de vida dos cidadãos. Tem-se por letramento em saúde o conhecimento e as competências pessoais adquiridas por meio das atividades diárias, interações sociais e gerações, as quais possibilitam às pessoas o acesso, compreensão, avaliação e utilização das informações e serviços, de modo que se mantenha e promova o bem-estar e a boa saúde para eles próprios e aqueles de seu convívio. O conhecimento e as competências pessoais são intermediados pelas estruturas organizacionais e pela disponibilidade de recursos^1^.

Estudos apontam para a necessidade de reconhecer o letramento em saúde como algo que transcende o indivíduo. Trata-se da compreensão de que as habilidades pessoais necessárias para lidar com questões de saúde são influenciadas pelo ambiente em que são aplicadas. Portanto, deve-se considerar a interação entre a complexidade e as demandas do sistema de saúde e as habilidades dos indivíduos^2-3^.

O inadequado letramento em saúde é considerado um problema de saúde pública. Dessa forma, é relevante reconhecer as condições de letramento em saúde da população, para que se possibilite o aprimoramento da educação e planejamento em saúde pública^2^. Os cuidadores de crianças com doenças crônicas complexas representam um conjunto crítico entre a população, pois além de serem responsáveis por sua própria vida e saúde, também devem zelar pela saúde e bem-estar dessas crianças sob sua responsabilidade^4-5^.

O *Maternal and Child Health Bureau* (MCHB) dos Estados Unidos da América (EUA) denominou essa população como *Children with Special Health Care Needs* (CSHCN), que no Brasil recebe a denominação de crianças com necessidades especiais de saúde (CRIANES)^6^. Atualmente, a terminologia “crianças que necessitam de atenção especial à saúde”, adotada neste estudo, tem sido empregada com base em um movimento epistemológico que compreende a necessidade como inerente à condição de saúde, e não à criança em si, conforme indicam estudos contemporâneos^7-8^. Tais crianças têm alto risco de apresentar ou já possuem condições crônicas, físicas, de desenvolvimento, comportamental ou emocional, necessidade de cuidados especiais e permanentes para manutenção de sua saúde, maior utilização dos serviços de saúde e atendimento de diferentes profissionais de múltiplas especialidades, além do exigido habitualmente pelas crianças^6,9^.

Diante dessas condições, essas crianças dependem fortemente das habilidades dos seus cuidadores quanto à compreensão e avaliação crítica das informações sobre saúde, comunicação com os profissionais de saúde e para navegar no sistema de saúde^10^. Assim, para a avaliação das condições do letramento em saúde de cuidadores de CRIANES é imprescindível a utilização de um instrumento abrangente que incorpore a multidimensionalidade do letramento em saúde^11-12^.

Cuidadores de crianças que necessitam de atenção especial à saúde enfrentam desafios diários para garantir um cuidado seguro e adequado, exigindo habilidades para compreender e aplicar informações sobre saúde. No entanto, barreiras relacionadas ao letramento em saúde, como dificuldades na obtenção e interpretação de informações médicas, podem comprometer a tomada de decisões, a adesão ao tratamento e a segurança da criança^4^.

Assim, identificar as condições do letramento em saúde de uma população fornece informações úteis e necessárias às organizações e profissionais de saúde para aprimorar as ações e serviços de saúde^13^. Estudos internacionais trazem evidências crescentes sobre a relação entre o inadequado letramento em saúde do cuidador e a falha na gestão do cuidado no domicílio e piora dos resultados de saúde dessas crianças, estando relacionado à baixa adesão ao tratamento, erros na administração de medicamentos no domicílio e maior número de hospitalizações não programadas^14-17^. Porém, no Brasil, esse cenário é pouco conhecido, carece de estudos que demonstrem as condições de letramento em saúde dessa população, justificando a necessidade deste estudo.

Este estudo busca preencher essa lacuna ao identificar as limitações e potencialidades dos cuidadores na navegação pelo sistema de saúde, na obtenção e aplicação de informações e na tomada de decisões para o cuidado dessas crianças. Para tanto, o estudo teve como objetivo explorar o letramento em saúde dos cuidadores de crianças que necessitam de atenção especial à saúde, identificando limitações e potencialidades e a relação com variáveis socioeconômicas, fontes de informação e suporte social.

## Método

### Delineamento do estudo

Trata-se de um estudo quantitativo, transversal, descritivo e analítico^18^, relatado conforme as diretrizes do *Strengthening the Reporting of Observational Studies in Epidemiology* (STROBE)^19^.

### Local e período

O estudo foi realizado no período entre dezembro/2022 e abril/2023 em Porto Alegre-RS, Brasil. Desenvolvido nas unidades pediátricas de um hospital de alta complexidade. O serviço de pediatria conta com 131 leitos, é referência nacional para atendimento de crianças portadoras de doenças crônicas de média e alta complexidade como, doenças gastrointestinais, genéticas, oncológicas, hematológicas, neurológicas, doença hepática, candidatos a transplante hepático e transplantados, transplante de células-tronco hematopoiéticas, entre outras doenças crônicas.

### População

Cuidadores principais das crianças que necessitam de atenção especial à saúde, hospitalizadas nas unidades pediátricas.

### Critérios de seleção e definição da amostra

Por cuidador principal, compreende-se um único indivíduo que se identifica como o responsável pela criança e por exercer os cuidados diários de forma permanente ou parcial, não remunerada e que não necessariamente possui laços consanguíneos, mas sim emocionais. Considerou-se criança o indivíduo que tinha idade inferior a 12 anos no período da coleta dos dados^20^. Para definição de elegibilidade como CRIANES, definiu-se que devam estar presentes ao menos em um domínio indicado através da aplicação da versão brasileira do instrumento *Children with Special Health Care Needs Screener* (CSHCN *Screener*®) dentre os três existentes, quais sejam, uso de medicamentos, uso de serviços de saúde e limitações funcionais^21^.

Inicialmente foram realizadas consultas nos prontuários eletrônicos dos pacientes hospitalizados nas unidades pediátricas para identificar aqueles menores de 12 anos. Posteriormente, a fim de rastrear as CRIANES, foi aplicado com o cuidador principal a versão brasileira do instrumento CSHCN *Screener*®^21^. Aos cuidadores principais cujas crianças foram identificadas como CRIANES, no mesmo momento, aplicaram-se os instrumentos de caracterização do cuidador e o questionário de avaliação do letramento em saúde ― HLQ-Br.

Foram incluídos no estudo aqueles maiores de 18 anos, que falam o português brasileiro (idioma das versões validadas dos instrumentos) como sua primeira língua usual e que se identificaram como cuidador principal, na hospitalização e no domicílio, da CRIANES menor de 12 anos, a qual já havia tido pelo menos uma alta hospitalar para o domicílio. Não foram estabelecidos critérios de exclusão para a seleção da amostra.

O número mínimo de participantes calculado para a amostra foi de 124 cuidadores, considerando-se um acréscimo de 10% para possíveis perdas e recusas, totalizando 138 participantes. O cálculo considerou poder de 80%, nível de significância de 5% e de Cohen de 0,51^22^. Este cálculo foi realizado por meio da ferramenta PSS Health,versão *online* ^23^.

### Instrumentos de coleta de dados e as variáveis do estudo

Para identificar as CRIANES hospitalizadas, aplicou-se a versão brasileira do CSHCN *Screener*®. É um instrumento de triagem, elaborado originalmente na língua inglesa, traduzido e adaptado para o português brasileiro, que mostrou boa consistência interna. O instrumento consegue identificar e avaliar as demandas de cuidados da criança que necessita de atenção especial à saúde relacionada a uma condição crônica, independente da presença de um diagnóstico clínico^6,21^. O uso deste instrumento justificou-se por sua sensibilidade em identificar as múltiplas dimensões das necessidades de saúde da criança. Isso inclui o critério de risco aumentado para condições crônicas, conforme a definição original de *Children with Special Health Care Needs*, que considera condições com duração mínima de 12 meses. Embora as crianças da amostra já apresentassem condições crônicas estabelecidas, o instrumento foi aplicado para confirmar a elegibilidade no momento da coleta e identificar os domínios das necessidades de atenção especial à saúde, uma vez que se tratava de um estudo voltado para a avaliação do letramento em saúde dos cuidadores de crianças já identificadas com necessidade de atenção especial à saúde.

O CSHCN *Screener*® contém cinco itens e cada um possui dois subitens (a e b), exceto o item cinco, que possui somente um. Todas as opções de resposta são do tipo “sim” ou “não”. Os subitens são questionados mediante resposta afirmativa no respectivo item e buscam relações dessas afirmativas com a vigência de qualquer problema de saúde cuja duração real ou esperada seja de, no mínimo, doze meses. Qualquer um dos itens que obtiver resposta afirmativa, juntamente com seus respectivos subitens, já indica que a criança possui alguma necessidade de atenção especial à saúde. Os cinco itens são distribuídos em três domínios de necessidades de atenção especial à saúde que exigem atendimento especializado de serviços de saúde e educacional, quais sejam, uso de medicamentos, uso de serviços de saúde e limitação funcional^21^.

Aplicou-se o Questionário de Caracterização da CRIANES e do cuidador principal, desenvolvido para este estudo, considerando a relevância de conhecer os fatores socioeconômicos, demográficos e culturais da família e o histórico de saúde da criança para um diagnóstico acurado sobre o letramento em saúde do cuidador. O questionário contém as variáveis sobre: a caracterização da CRIANES ― origem das necessidades de atenção especiais à saúde (causa congênita, adquirida e diagnóstico médico), categorias das demandas de cuidados (desenvolvimento, tecnológicos, medicamentosos, habituais modificados, mistos e clinicamente complexos), motivo da internação atual, data da internação e de nascimento, sexo, motivo pelo qual e a data da re-internação após a primeira alta hospitalar, número de idas à emergência ou internações hospitalares no último ano e o motivo, utilização de outros serviços de saúde; e a caracterização socioeconômica do cuidador ― grau de parentesco com a CRIANES, data de nascimento, sexo, escolaridade, profissão/ocupação, renda familiar, possui outros filhos, outras pessoas que necessitam de atenção especial à saúde dependente dos seus cuidados, principais fontes de informação de saúde (televisão, jornal, rádio, *internet* e outros), cidade e estado de residência e existência de outro cuidador principal.

Para avaliar as condições do letramento em saúde dos cuidadores principais, aplicou-se a versão brasileira do *Health Literacy Questionnaire* (HLQ-Br). O *Health Literacy Questionnaire* é um instrumento multidimensional que foi traduzido para o português brasileiro e validado com 794 adultos usuários do Sistema Único de Saúde (SUS) de três regiões distintas do Brasil. Neste processo de validação, obteve boas propriedades psicométricas. É um instrumento multidimensional, possui 44 itens distribuídos em nove escalas que são subdivididas em duas partes. A Parte 1 tem quatro opções de respostas entre “discordo totalmente” e “concordo totalmente” (escores 1 - 4), a Parte 2 tem cinco opções de respostas entre “não consigo fazer ou sempre difícil” e “sempre fácil” (escores 1-5). Este instrumento não fornece pontuação global, e sim escores com a média individual das nove escalas. A pontuação mais próxima do limite superior ou inferior indica, respectivamente, as potencialidades e as limitações dos cuidadores nos cuidados em relação à sua saúde^24^.

Para que a versão brasileira do HLQ pudesse ser aplicada a este estudo, obteve-se a autorização de uso pela Universidade de Deakin, Austrália, pelo endereço de e-mail “HLQ-info@swin.edu.au”.

### Coleta dos dados

A coleta de dados se deu no prontuário eletrônico do paciente e por meio de entrevista com o cuidador principal da CRIANES, realizada em sala reservada e com privacidade nas próprias unidades pediátricas. A equipe de pesquisadores foi previamente capacitada para a coleta de dados nos prontuários e para a aplicação dos questionários.

### Tratamento e análise dos dados

Os dados foram analisados pelo programa *Statistical Package for the Social Sciences* (SPSS/PASW), versão 27.0. Na análise descritiva das variáveis quantitativas utilizou-se média e desvio padrão e para a análise das variáveis categóricas foram utilizadas frequências absolutas e relativas. A decisão pelo tipo de distribuição foi baseada no tamanho do desvio padrão (não podendo ultrapassar a metade da média para considerar a distribuição simétrica) e na proximidade entre média e mediana (diferença inferior a 20%). O nível de significância adotado foi de 5% (p≤ 0,05).

Para comparar médias, foram aplicados o teste t-Student ou a Análise de Variância (ANOVA) em conjunto com o teste de Tukey. Ainda, para avaliar a associação entre as variáveis categóricas, os testes Qui-quadrado de Pearson ou exato de Fisher foram aplicados. Já para avaliar a associação entre as variáveis ordinais com os escores do HLQ- Br, foi utilizado o teste da correlação de Spearman.

### Aspectos éticos

Este estudo teve seu projeto aprovado pelo Comitê de Ética em Pesquisa da instituição em estudo sob o parecer n.º 5.751.478. Os aspectos éticos foram respeitados e seguidos de acordo com as legislações vigentes. Os participantes assinaram o Termo de Consentimento Livre e Esclarecido.

## Resultados

A amostra compreendeu 139 cuidadores principais de CRIANES hospitalizadas. A maioria desses cuidadores era do sexo feminino (89,9%), tinha idade entre 31 e 60 anos (62,6%), ensino médio completo (55%), era trabalhador do lar (79%), renda familiar de um a dois salários mínimos (73,4%) e era proveniente de diferentes municípios da região Sul do Brasil (95,6%).

Da amostra, 39 cuidadores (28,1%) tinham outro indivíduo dependente do seu cuidado que necessitava de atenção especial à saúde, além da CRIANES. Os cuidadores principais eram predominantemente mães (84,2%), as quais tinham outros filhos além da CRIANES (69,1%) e 86 (61,9%) cuidadores principais referiram ter o suporte de um segundo cuidador principal para os cuidados com a criança, e destes, 38,1% eram o pai.

Os participantes eram cuidadores principais de crianças que necessitavam de atenção especial à saúde relacionada ao uso de medicamentos por uma condição crônica (94,2%), uso de serviços de saúde maior do que a maioria das crianças da mesma idade (100%) e tinham alguma limitação funcional ou incapacidade para realizar atividades comuns à maioria das crianças (53,2%). Sendo que 137 (97,8%) dessas crianças tinham mais de uma necessidade de atenção especial à saúde e 98 (70,5%) eram também crianças dependentes de tecnologias que faziam uso de pelo menos um dispositivo médico.

Na análise de grupos das combinações dos domínios de necessidades de atenção especial à saúde das CRIANES, apenas três (2,2%) necessitavam somente de serviços de saúde (SS); cinco (3,6%) necessitavam de uso de Serviços de Saúde (SS) e tinham Limitação Funcional (LF), enquanto 62 (44,6%) faziam Uso de Medicamentos e SS e a maioria (69 – 49,6%) das CRIANES tinha LF e necessitava de Uso de Medicamentos e de SS.

Conforme descrito na Tabela 1, considerando os escores médios das nove escalas do questionário HLQ-Br, foram identificadas limitações no letramento em saúde dos cuidadores em relação a Cuidado ativo da saúde (Escala 3 – Parte 1), Suporte social para saúde (Escala 4 - Parte 1) e Capacidade de encontrar boas informações sobre saúde (Escala 8 - Parte 2). Potencialidades no letramento em saúde dos cuidadores foram identificadas no que se refere à Avaliação das informações sobre saúde (Escala 5 – Parte 1) e Capacidade de interagir ativamente com os profissionais de saúde (Escala 6 – Parte 2).

**Tabela 1-:** Escores médios das escalas e dos itens do HLQ-Br* que indicam maiores limitações e potencialidades do letramento em saúde dos cuidadores principais de CRIANES† hospitalizadas (n = 139). Porto Alegre, RS, Brasil, 2023

**Escalas e questões do HLQ-Br** *	**Média**	**Desvio-Padrão**
**Parte 1 (Escores 1-4)**		
**Escala 1 – Compreensão e apoio dos profissionais da saúde**	3,02	0,53
Q ^‡^ 17 – Eu tenho os profissionais de saúde que necessito para…	3,25	0,71
Q ^‡^ 8 – Eu tenho pelo menos um profissional de saúde com quem…	2,79	0,91
**Escala 2 – Informações suficientes para cuidar da saúde**	3,03	0,47
Q ^‡^ 23 – Eu tenho toda a informação que preciso para…	3,68	0,64
Q ^‡^ 14 – Eu tenho certeza de que tenho toda informação que…	3,01	0,73
**Escala 3 – Cuidado ativo da saúde**	2,82	0,51
Q ^‡^ 6 – Eu gasto bastante tempo envolvido com…	3,40	0,95
Q ^‡^ 21 - Há coisas que eu faço regularmente para…	2,51	0,70
**Escala 4 – Suporte social para saúde**	2,96	0,59
Q ^‡^ 19 – Eu tenho forte apoio…	3,24	0,76
Q ^‡^ 5 – Quando me sinto doente, as pessoas ao meu redor…	2,75	0,78
**Escala 5 – Avaliação das informações em saúde**	3,13	0,45
Q ^‡^ 7 – Quando eu vejo novas informações sobre saúde…	3,37	0,62
Q ^‡^ 4 – Eu comparo informações de saúde obtidas…	2,94	0,76
**Parte 2 (Escores 1 – 5)**		
**Escala 6 – Capacidade de interagir ativamente com os profissionais de saúde**	4,00	0,57
Q ^‡^ 20 – Fazer perguntas aos profissionais de saúde para…	4,39	0,83
Q ^‡^ 2 – Ter certeza de que os profissionais de saúde…	3,60	0,96
**Escala 7 – Navegar no sistema de saúde**	3,70	0,74
Q ^‡^ 13 – Ter certeza de encontrar o lugar correto para…	4,01	1,07
Q ^‡^ 1 – Encontrar o serviço de saúde…	3,31	1,08
**Escala 8 – Capacidade de encontrar boas informações sobre saúde**	3,60	0,64
Q ^‡^ 10 – Conseguir informação sobre saúde para…	3,81	0,91
Q ^‡^ 18 – Conseguir informações sobre…	3,37	1,19
**Escala 9 – Compreender as informações sobre saúde e saber o que fazer**	3,99	0,57
Q ^‡^ 21 – Entender o que os profissionais de saúde estão…	4,38	0,71
Q ^‡^ 12 – Ler e entender informações…	3,81	1,06

*HLQ-Br = Versão brasileira do *Health Literacy Questionnaire*; ^†^CRIANES = Crianças que necessitam de atenção especial à saúde; ^‡^Q = Questão

Todos os cuidadores participantes passaram por, no mínimo, uma hospitalização com a CRIANES nos últimos 12 meses que antecederam a coleta de dados deste estudo. Destes, 30 (21,6%) internaram apenas uma vez, 74 (53,2%) internaram de duas a seis vezes, 23 (16,5%) internaram de sete a onze vezes e 12 (8,7%) foram hospitalizados doze ou mais vezes no período.

Na Tabela 2 estão descritos os coeficientes de correlação entre os escores médios das nove escalas do questionário HLQ-Br e as variáveis médias do número de hospitalizações das CRIANES, grau de instrução e renda familiar dos cuidadores principais. As associações positivas e estatisticamente significativas (p<0,05) indicam a relação entre o grau de instrução e condições de renda familiar dos cuidadores com as suas limitações e potencialidades do letramento em saúde. Assim como, em relação ao número de hospitalizações necessárias à CRIANES.

**Tabela 2-:** Correlação entre os escores médios das escalas do HLQ-Br* com as características socioeconômicas dos cuidadores principais de CRIANES^†^e o número de hospitalizações dessas crianças (n = 139). Porto Alegre, RS, Brasil, 2023

**Escalas HLQ-Br** *	**Grau de instrução**	**Renda familiar**	**Hospitalizações da CRIANES** ^†^
**Parte 1**			
**1 -** Compreensão e apoio dos profissionais de saúde	r _s_^‡^ =0,23 ^§^	r _s_^‡^ =0,23 ^§^	r _s_^‡^ =0,16
	(p=0,006)	(p=0,006)	(p=0,057)
**2 -** Informações suficientes para cuidar da saúde	r _s_^‡^ =0,15	r _s_^‡^ =0,09	r _s_^‡^ =0,17
	(p=0,084)	(p=0,302)	(p=0,053)
**3 -** Cuidado ativo da saúde	r _s_^‡^ =0,10	r _s_^‡^ =0,09	r _s_^‡^ =0,08
	(p=0,260)	(p=0,292)	(p=0,334)
**4 -** Suporte social para saúde	r _s_^‡^ =0,33 ^§^	r _s_^‡^ =0,32 ^§^	r _s_^‡^ = - 0,004
	(p=0,000)	(p=0,000)	(p=0,96)
**5 -** Avaliação das informações em saúde	r _s_^‡^ =0,17 ^||^	r _s_^‡^ =0,17 ^||^	r _s_^‡^ =0,19 ^||^
	(p=0,038)	(p=0,044)	(p=0,019)
**Parte 2**			
**6 -** Capacidade de interagir ativamente com os profissionais de saúde	r _s_^‡^ =0,13	r _s_^‡^ =0,15	r _s_^‡^ = - 0,12
	(p=0,115)	(p=0,064)	(p=0,158)
**7 -** Navegar no sistema de saúde	r _s_^‡^ = 0,62	r _s_^‡^ =0,20 ^||^	r _s_^‡^ = - 0,051
	(=0,467)	(p=0,016)	(p=0,553)
**8 -** Capacidade de encontrar boas informações sobre saúde	r _s_^‡^ =0,35 ^§^	r _s_^‡^ =0,26 ^§^	r _s_^‡^ = - 0,01
	(p=0,000)	(p=0,002)	(p=0,837)
**9 -** Compreender as informações sobre saúde e saber o que fazer	r _s_^‡^ =0,22 ^§^	r _s_^‡^ =0,16 ^||^	r _s_^‡^ =0,01
	(p=0,009)	(p=0,049)	(p=0,838)

*HLQ-Br = Versão brasileira do *Health Literacy Questionnaire*; ^†^CRIANES = Crianças que necessitam de atenção especial à saúde; ^‡^r_s_ = Coeficiente de correlação de Spearman; ^§^Correlação significativa no nível 0,01; ^||^Correlação significativa no nível 0,05

Em relação à parte 1 do questionário HLQ-Br, houve associação positiva e estatisticamente significativa entre as variáveis grau de instrução e renda familiar com as escalas 1 - Compreensão e apoio dos profissionais de saúde (p = 0,006), 4 – Suporte social para saúde (p = 0,000) e 5 - Avaliação das informações em saúde (p = 0,038; p = 0,044). Ainda, houve associação positiva e significativa entre a variável hospitalizações da CRIANES com a escala 5 – Avaliação das informações em saúde (p = 0,019) (Tabela 2).

Na Parte 2, identificou-se associação positiva e estatisticamente significativa entre as variáveis grau de instrução e renda familiar com as escalas 8 – Capacidade de encontrar boas informações sobre saúde (p = 0,000 - p = 0,002) e 9 – Compreender as informações sobre saúde e saber o que fazer (p = 0,009 - p = 0,049). Ainda, constatou-se a associação entre a variável renda familiar e a escala 7 – Navegar no sistema de saúde (p = 0,016) (Tabela 2).

A maioria dos cuidadores referiu ter a *internet* (87%) como uma de suas principais fontes de informações sobre saúde, enquanto os profissionais de saúde foram referidos como principal fonte por 19 cuidadores (13,6%) e três (2,1%) apontaram outros cuidadores principais como sendo uma de suas principais fontes de informação sobre saúde.

Potencialidades no letramento em saúde quanto à “Compreensão e apoio dos profissionais de saúde (Escala 1)” foram identificadas naqueles cuidadores que referiram ter os profissionais da saúde como uma de suas principais fontes de informação sobre saúde (p = 0,002). Já em relação à “Avaliação das informações em saúde (Escala 5)”, observaram-se potencialidades no letramento em saúde daqueles cuidadores que apontaram a *internet* (p = 0,026) como uma das principais fontes de informação sobre saúde (Tabela 3).

Limitações no letramento em saúde quanto ao “Suporte social para saúde (Escala 4)” e à “Avaliação das informações em saúde (Escala 5)” foram identificadas naqueles cuidadores que referiram não ter o suporte de um segundo cuidador principal para com a CRIANES (p = 0,039 - p = 0,037), conforme a comparação das médias descritas na Tabela 3.

**Tabela 3-:** Associação das condições de letramento em saúde dos cuidadores principais de CRIANES* conforme o questionário HLQ-Br^†^e as variáveis rede de apoio para o cuidado e as fontes de informação sobre saúde (n = 139). Porto Alegre, RS, Brasil, 2023

**Escalas HLQ-Br** ^†^	**E** ^‡^ **1**	**E** ^‡^ **2**	**E** ^‡^ **3**	**E** ^‡^ **4**	**E** ^‡^ **5**	**E** ^‡^ **6**	**E** ^‡^ **7**	**E** ^‡^ **8**	**E** ^‡^ **9**
**Variáveis**	Méd. ^§^ ± DP ^||^	Méd. ^§^ ± DP ^||^	Méd. ^§^ ± DP ^||^	Méd. ^§^ ± DP ^||^	Méd. ^§^ ± DP ^||^	Méd. ^§^ ± DP ^||^	Méd. ^§^ ± DP ^||^	Méd. ^§^ ± DP ^||^	Méd. ^§^ ± DP ^||^
**FI** ^¶ -^ **Outro cuidador**									
Não	2,97±0,51	3,01±0,52	2,73±0,53	2,83±0,58	3,03±0,43	4,04±0,54	3,74±0,76	3,58±0,59	4,04±0,50
Sim	3,06±0,55	3,04±0,44	2,88±0,49	3,05±0,59	3,10±0,45	3,97±0,59	3,68±0,73	3,60±0,67	3,97±0,60
p- *value* **	0,366	0,750	0,086	0,039	0,037	0,448	0,618	0,855	0,489
**FI** ^¶^ **- *Internet***									
Não	2,87±0,44	3,06±0,49	2,93±0,49	2,97±0,56	2,91±0,51	4,00±0,61	3,96±0,93	3,55±0,74	4,10±0,54
Sim	3,05±0,54	3,02± 0,47	2,81±0,51	2,96±0,59	3,16±0,43	4,00±0,57	3,66±0,70	3,60±0,62	3,98±0,57
p- *value* **	0,192	0,711	0,349	0,925	0,026	0,99	0,115	0,74	0,42
**FI** ^¶^ **- Jornal/livro**									
Não	3,02±0,52	3,02±0,46	2,83±0,50	2,97±0,58	3,10±0,44	4,00±0,58	3,71±0,74	3,57±0,64	3,99±0,56
Sim	3,04±0,62	3,04±0,56	2,780,67	2,83±0,62	3,40±0,47	4,00±0,56	3,63±0,77	3,90±0,47	4,03±0,66
p- *value* **	0,915	0,914	0,760	0,452	0,041	0,993	0,751	0,098	0,820
**FI** ^¶^ **- TV/Rádio**									
Não	3,06±0,53	3,04±0,46	2,83±0,49	2,97±0,59	2,94±0,59	3,15±0,42	3,74±0,69	3,60±0,61	4,03±0,50
Sim	2,91±0,523	2,98±0,49	2,80±0,56	2,94±0,59	3,07±0,52	3,80±0,56	3,60±0,85	3,59±0,72	3,88±0,71
p- *value* **	0,14	0,511	0,707	0,815	0,333	0,013	0,335	0,940	0,165
**FI** ^¶^ **- Prof. Saúde**									
Não	2,97±0,52	3,01±0,49	2,85±0,50	2,98±0,58	3,11±0,45	4,00±0,58	3,72±0,75	3,61±0,65	4,01±0,58
Sim	3,38±0,49	3,13±0,30	2,66±0,52	2,87±0,62	3,24±0,46	3,95±0,55	3,57±0,63	3,50±0,55	3,90±0,48
p- *value* **	**0,002**	0,320	0,134	0,468	0,458	0,725	0,397	0,484	0,446
**FI** ^¶^ **– Cuidadores**									
Não	3,02±0,53	3,03±0,47	2,84±0,50	2,97±0,59	3,12±0,45	4,00±0,58	3,71±0,74	3,60±0,64	3,99±0,57
Sim	3,25±0,47	2,91±0,62	2,13±0,46	2,60±0,34	3,40±0,52	4,06±0,41	3,22±0,63	3,53±0,46	4,00±0,52
p- *value* **	0,47	0,676	0,017	0,280	0,304	0,844	0,257	0,853	0,997

*CRIANES = Crianças que necessitam de atenção especial à saúde; ^†^HLQ-Br = Versão brasileira do *Health Literacy Questionnaire*; ^‡^E = Escala; ^§^Méd. = Média; ^||^DP = Desvio-padrão; ^¶^FI = Fonte de informação sobre saúde; **p-*value* = Teste t-Student

## Discussão

O estudo inova ao analisar as condições do letramento em saúde de cuidadores de CRIANES, utilizando-se de um instrumento multidimensional de avaliação. Resultando, assim, no reconhecimento das limitações e potencialidades quanto às habilidades funcionais, interativas e críticas desses cuidadores, associando-as com as suas condições socioeconômicas e as demandas de cuidados necessários às CRIANES^2^.

As CRIANES exigem elevada demanda de cuidados específicos e complexos, além disso, requerem tratamento e acompanhamento multiprofissional por longo período. A condução desse cenário complexo requer conhecimentos específicos, habilidades técnicas e de comunicação por parte do cuidador principal^25-26^.

Neste estudo, todas as CRIANES foram identificadas como dependentes dos serviços de saúde e apresentaram um número expressivo de hospitalizações no período de um ano. A maioria das CRIANES foi reconhecida na combinação dos três domínios de necessidades especiais de saúde, além de serem dependentes de dispositivos tecnológicos para manutenção da vida. Tais achados evidenciam a complexidade dos cuidados necessários a essas crianças e que frequentemente são demandados aos cuidadores no domicílio, sem haver um preparo para a desospitalização nem suporte adequado da rede de serviços de saúde^27-29^.

O cuidador principal é reconhecido como parte fundamental para o cuidado seguro e de qualidade da CRIANES no domicílio. Tem papel crucial na tomada de decisões, junto aos profissionais da saúde, referentes às necessidades de saúde dessa criança e, sobretudo, no gerenciamento e execução das demandas de cuidados no domicílio. Além dos desafios gerados pela complexidade dos cuidados, os cuidadores também enfrentam uma série de dificuldades impostas pelo próprio sistema de saúde^(15,30-31)^.

Para atender a essas demandas e superar as dificuldades impostas, o cuidador precisa desenvolver habilidades cognitivas e sociais que lhe possibilitem navegar no sistema de saúde, obter, compreender e aplicar as informações necessárias para gerenciar os cuidados, fazer julgamentos e tomar decisões acerca das necessidades e particularidades dessas crianças^26,32^.

Os resultados deste estudo demonstraram condições razoáveis de letramento em saúde dos cuidadores, reconhecidas nas escalas do questionário aplicado, ainda que limitações relacionadas às habilidades funcional, interativa e crítica também tenham sido identificadas^2^. Ressalta-se que o letramento em saúde de um indivíduo não é estático. Fatores situacionais como ambiente desconhecido, interação intimidante e fatores emocionais podem afetar temporária e negativamente as condições de letramento em saúde e a capacidade desses cuidadores de gerenciar os cuidados necessários à CRIANES^32-34^.

Há limitações em relação ao cuidado ativo da saúde por parte dos cuidadores deste estudo no que se refere às habilidades interativa e crítica. Esses cuidadores, ainda que tenham as habilidades e conhecimentos necessários para a prática do autocuidado, não agem em prol de promovê-lo, pois não o veem como prioridade. Achados semelhantes são identificados em outras pesquisas nas quais o cuidador principal se dedica exclusivamente à demanda intensiva de cuidados complexos exigidos pela CRIANES e acaba não se envolvendo com seu próprio cuidado, o que frequentemente resulta em sobrecarga física, emocional e social^(31,35-37)^.

Conforme o esperado, as mães identificaram-se, majoritariamente, como sendo o cuidador principal da CRIANES na hospitalização e no domicílio, como já identificado globalmente em outras pesquisas^(25-26,34,38)^. Os determinantes sociais que apresentaram relação significativa com a condição de letramento em saúde desses cuidadores foram grau de instrução e renda familiar, assim como identificado em outros estudos^(17,39-41)^.

A carência de suporte social também expõe os cuidadores à sobrecarga física e mental, podendo afetar sua saúde, bem-estar e qualidade de vida, além de dificultar a prática de habilidades já adquiridas de letramento em saúde e alterar a capacidade de execução dos cuidados com a CRIANES^33,37^. Neste estudo, a maioria dos cuidadores referiu ter amplo suporte da rede de apoio primária, contando inclusive com o auxílio de um segundo cuidador para revezamento dos cuidados, porém também referiram não se sentir compreendidas pelas pessoas próximas quando se sentem doentes.

Já em outro estudo, mães cuidadoras de CRIANES possuem limitado suporte social, relataram não poder contar com o suporte do pai da criança para realizar os cuidados no domicílio e sentem grande desgaste mental e físico. Quanto ao relacionamento social familiar, elas referem sentimentos de isolamento, incompreensão, falta de empatia e dificuldade de comunicação. Embora não recebam o suporte familiar adequado, por meio do compartilhamento de experiências, essas cuidadoras vivenciam fortes vínculos com outras mães de CRIANES, pelas quais se sentem compreendidas e apoiadas^42^.

Receber o suporte social de amigos e familiares tem sido apontado como essencial para o manejo das necessidades da criança, compartilhamento dos cuidados e, sobretudo, para a incorporação de novas informações sobre os desafios diante da complexidade de cuidar de uma CRIANES. As condições mais avançadas de habilidades interativas e comunicativas críticas favorecem o fortalecimento das redes de apoio primária e secundária e possibilitam maior extração de informações de diferentes fontes^32,37^.

A rede social secundária fragilizada também contribui para limitações no letramento em saúde de cuidadores. Estudos identificam as instituições de saúde e assistência social como rede de apoio secundária, descrevem o vínculo entre cuidadores e profissionais da saúde como conflituoso, frágil ou rompido. A fragilidade desse vínculo é associada à fragmentação na organização dos serviços de saúde, pouca qualidade da assistência, limitada responsabilidade por parte dos profissionais da saúde, lacunas na comunicação, insuficiência de orientação e distanciamento com o cuidador^37,43^.

Tratando-se de cuidadores de CRIANES, a falta de habilidades para encontrar informações e navegar no sistema de saúde por conta própria constitui uma barreira de grande relevância para o cuidado no domicílio e indica limitações no letramento em saúde funcional e interativo desses cuidadores^32^. Essas crianças necessitam do uso frequente dos serviços de saúde e de profissionais de múltiplas especialidades. Quanto maior a equipe de profissionais, mais visitas aos serviços de saúde o cuidador precisa gerenciar, exigindo habilidades avançadas de comunicação e coordenação^9,38^.

Dificuldades na navegação são frequentemente causadas pelo próprio sistema de saúde. Estudos que avaliam o itinerário terapêutico de CRIANES identificam o despreparo dos profissionais de saúde e a fragmentação, desarticulação, falta de estrutura e precariedade dos serviços de saúde como barreiras para acesso e navegação na rede de serviços de saúde^(31,44-45)^. A comunicação entre profissionais de saúde responsáveis pelo tratamento da criança é percebida por cuidadores como escassa e insuficiente, assim como a comunicação de profissionais com o cuidador^37^.

Para atender às demandas de cuidados exigidas pelas CRIANES, o cuidador precisa ter acesso a uma gama de informações específicas e muitas vezes complexas. Diante das fragilidades de comunicação, preparo e orientação para a transição do cuidado por parte dos profissionais e serviços de saúde^46^, a incapacidade de conseguir acesso às informações sobre saúde por conta própria se torna ainda mais preocupante.

Neste estudo, a maioria dos cuidadores reconheceu a *internet* como uma das principais fontes de informação sobre saúde e raros foram os cuidadores que identificaram os profissionais de saúde neste papel. O reconhecimento das redes digital e social, assim como dos profissionais da saúde como fontes de informação pelos cuidadores de CRIANES também foi descrito em outros estudos, nos quais se valorizam as informações dos profissionais, porém com orientações pouco compatíveis e aplicáveis à realidade da família e da criança. Nesses casos, buscou-se a troca de experiências com outros cuidadores de crianças com necessidades iguais ou semelhantes^47^. Assim como neste estudo, também se reconheceram outros cuidadores de CRIANES como sendo importante fonte de informação, troca de experiências e alternativas para qualificar o cuidado à CRIANES.

Em outro estudo, identificou-se que cuidadores de crianças com doenças crônicas são mais propensos a acessar redes sociais que discutem a saúde da criança, apesar de classificá-las como as fontes menos confiáveis de informações online sobre saúde. Ainda, ao encontrar informações que contradiziam o conselho do profissional, a maioria dos pais relatou seguir o conselho do profissional ou consultá-lo antes de tomar uma decisão^48^.

Nesta pesquisa, foram identificadas potencialidades no letramento em saúde interativo dos cuidadores em relação à capacidade de interagir ativamente com os profissionais da saúde. Apesar de a maioria não identificar o profissional de saúde como fonte de informação sobre saúde, sentem-se capazes de procurar orientações adicionais com esses profissionais, fazendo perguntas para obter as informações de que necessitam. Atribuiu-se esse resultado à longa experiência e convivência desses cuidadores com profissionais e serviços de saúde, devido ao frequente uso dos mesmos serviços para atendimento das demandas de cuidados da CRIANES^37^.

Potencialidades também foram reconhecidas no letramento em saúde crítico dos cuidadores quanto à habilidade de avaliação das informações em saúde, inclusive identificando associação estaticamente significativa entre a escala correspondente ao item do questionário aplicado com aqueles cuidadores que utilizam a *internet* como fonte de informação sobre saúde.

Ressalta-se que a *internet* pode ser um instrumento valioso de comunicação e de aquisição de informações sobre saúde para esses cuidadores, desta forma desenvolvendo a capacidade de interação social, maior conhecimento sobre saúde, melhorando a autonomia e autoconfiança para o gerenciamento dos cuidados. Ao mesmo tempo, pode tornar-se também um grande risco para a saúde tanto dos cuidadores quanto das crianças, já que se trata de uma fonte de informação muitas vezes insegura e incompleta. É necessário que o cuidador tenha a capacidade de identificar boas e confiáveis fontes de informação e de distinguir informações contraditórias^(46,49-50)^. Diante deste cenário, torna-se relevante a avaliação do letramento digital em saúde dessa população.

Este estudo se destaca por dar voz aos cuidadores de CRIANES, reconhecendo seus desafios diários na busca por informações e na tomada de decisões para garantir o bem-estar de suas crianças. Utilizando instrumentos validados internacionalmente, oferece um retrato fiel da realidade desses cuidadores, trazendo um olhar inédito para essa questão no Brasil. Além de contribuir cientificamente, reforça a necessidade de aproximar os profissionais de saúde das famílias, fortalecendo o letramento em saúde e a autonomia desses cuidadores.

As limitações deste estudo relacionam-se à coleta de dados realizada em apenas uma instituição de saúde, o que pode não representar a realidade de cuidadores em outros contextos, como aqueles que acessam serviços de menor complexidade ou que enfrentam diferentes desafios regionais no sistema de saúde, além da ausência de uma abordagem qualitativa que explore as causas subjacentes às condições multidimensionais do letramento em saúde dos cuidadores. Além disso, o estudo foi transversal, não permitindo análise de mudanças no letramento em saúde.

Portanto, sugerem-se novos estudos em mais instituições de saúde e de abordagem mista e longitudinais, o que permitiria o desenvolvimento de intervenções amplas e personalizadas para atender às necessidades de letramento em saúde de cuidadores de CRIANES.

## Conclusão

O estudo inova ao explorar o letramento em saúde multidimensional de cuidadores de CRIANES no âmbito nacional, utilizando-se da associação de instrumentos robustos e amplamente aplicados internacionalmente, tanto para o rastreio e classificação das CRIANES em domínios de necessidades, quanto para a avaliação das condições de letramento em saúde do cuidador principal para além de apenas uma capacidade ou limitação cognitiva.

Os achados revelam a influência do grau de instrução do cuidador e da renda familiar nas condições do letramento em saúde, além de identificar a *internet* como principal fonte de informação desses cuidadores. Esses, em sua maioria, possuem capacidade limitada de agir em prol da sua própria saúde e bem-estar e dispõem de limitado suporte social, apesar de contarem com o auxílio de um segundo cuidador para atender às necessidades da CRIANES. Além disso, sentem-se pouco compreendidos e sem apoio das pessoas próximas quando se trata da sua própria condição de saúde. E ainda, possuem limitada capacidade de encontrar boas informações sobre saúde por conta própria.

Ressalta-se a relevância do reconhecimento das limitações e potencialidades no letramento em saúde dessa população como um recurso importante que permite a instrumentalização para a realização de intervenções educacionais assertivas por parte dos serviços e profissionais da saúde que têm como responsabilidade serem promotores do letramento em saúde, fortalecendo o cuidado ativo, melhorando o acesso, compreensão e uso das orientações, aumentando a qualidade e segurança do cuidado.

## Data Availability

Os conjuntos de dados relacionados a este artigo estarão disponíveis mediante solicitação ao autor correspondente.

# Translation: Alfabetización en salud de cuidadores primarios de niños que requieren cuidados especiales de salud: estudio transversal*

**DOI:** 10.1590/1518-8345.7860.4688

**Authors:** Sara Raquel Kuntz, Katarinne Lima Moraes, Alessandra Vaccari, Thailine Stodulski Theis, Ana Luísa Petersen Cogo, Wiliam Wegner

**Affiliations:** 1 Universidade Federal do Rio Grande do Sul, Escola de Enfermagem, Porto Alegre, RS, Brasil.; 2 Hospital de Clínicas de Porto Alegre, Serviço de Enfermagem Pediátrica, Porto Alegre, RS, Brasil.; 3 Universidade de Brasília, Faculdade de Ceilândia, Brasília, DF, Brasil.; 4 Becaria del Conselho Nacional de Desenvolvimento Científico e Tecnológico (CNPq), Brasil.

**Keywords:** Alfabetización en Salud, Niño, Enfermedad Crónica, Cuidadores, Enfermería Pediátrica, Seguridad del Paciente

## Abstract

explorar la alfabetización en salud de los cuidadores primarios de niños que requieren cuidados especiales de salud, e identificar limitaciones, potencialidades y la relación con variables socioeconómicas, fuentes de información y apoyo social.

estudio transversal, descriptivo y analítico, realizado con 139 cuidadores. Los datos fueron recolectados entre diciembre de 2022 y abril de 2023, en un hospital del sur de Brasil, a través del *Children with Special Health Care Needs Screener, del Health Literacy Questionnaire* y de un cuestionario de caracterización. El análisis incluyó estadísticas descriptivas e inferenciales.

se identificaron limitaciones en la atención activa, el apoyo social y la capacidad para encontrar buena información de salud. Se observaron potencialidades en la evaluación de la información y en la interacción con los profesionales de la salud. Se encontró asociación positiva entre las variables educación e ingreso familiar con las escalas comprensión y apoyo profesional en salud (*p*=0,006), apoyo social a la salud (*p*=0,000) y evaluación de la información en salud (*p*=0,038).

las intervenciones para cuidadores deben fortalecer la atención activa, el apoyo social y el acceso a la información, considerando la influencia de los factores socioeconómicos y la importancia de la comunicación con los profesionales para mejorar el acceso, la comprensión y el seguimiento de las orientaciones, aumentando la calidad y la seguridad de la atención.

## Destacados

**(1)** El nivel de educación y el ingreso familiar influyen en las condiciones de alfabetización en salud.**(2)** *Internet* como principal fuente de información sobre la salud de los cuidadores. **(3)** Capacidad limitada para encontrar buena información sobre salud por su cuenta. **(4)** Prestación de apoyo y soporte social limitado a cuidadores de NANEAS.

## Introducción

La alfabetización en salud es reconocida como un determinante social importante y modificable de la salud, advirtiéndose una relación con la calidad de vida de los ciudadanos. La alfabetización en salud es el conocimiento y las habilidades personales adquiridas a través de las actividades diarias, las interacciones sociales y las generaciones, que permiten a las personas acceder, comprender, evaluar y utilizar la información y los servicios, con el fin de mantener y promover el bienestar y la buena salud para sí mismas y para quienes las rodean. El conocimiento y las habilidades personales están mediadas por las estructuras organizacionales y la disponibilidad de recursos^1^.

Los estudios apuntan a la necesidad de reconocer la alfabetización en salud como algo que trasciende al sujeto. Esto implica comprender que las habilidades personales necesarias para abordar problemas de salud están influenciadas por el entorno en el que se aplican. Por lo tanto, debe considerarse la interacción entre la complejidad y las demandas del sistema de salud y las capacidades de las personas^2-3^.

La alfabetización sanitaria inadecuada se considera un problema de salud pública. En ese marco, es importante reconocer las condiciones de alfabetización en salud de la población, para posibilitar el mejoramiento de la educación y la planificación en materia de salud pública^2^. Los cuidadores de niños con enfermedades crónicas complejas representan un grupo crítico dentro de la población, ya que además de ser responsables de su propia vida y salud, también deben velar por la salud y el bienestar de los niños bajo su responsabilidad^4-5^.

La *Maternal and Child Health Bureau* (MCHB) de Estados Unidos ha denominado a esta población como *Children with Special Health Care Needs* — CSHCN, que en Brasil se denomina *Crianças com Necessidades Especiais de Saúde* (CRIANES)^6^. Actualmente, la terminología “niños que requieren cuidados especiales de salud”, adoptada en este estudio, ha sido utilizada con base en un movimiento epistemológico que entiende la necesidad como inherente a la condición de salud, y no al niño en sí, como indican estudios contemporáneos^7-8^. Existe un alto riesgo de que estos niños presenten o ya padezcan condiciones físicas crónicas, del desarrollo, conductuales o emocionales, y requieran de cuidados especiales y permanentes para la conservación de su salud, mayor utilización de los servicios de salud y atención de diferentes profesionales de múltiples especialidades, además del cuidado que habitualmente requieren los niños^6,9^.

Dadas estas condiciones, estos niños dependen en gran medida de las habilidades de sus cuidadores para comprender y evaluar críticamente la información de salud, comunicarse con los profesionales de la salud y navegar por el sistema de salud^10^. En consecuencia, para evaluar las condiciones de alfabetización en salud de los cuidadores de NANEAS (Niños con Necesidades Especiales de Atención en Salud), es fundamental utilizar un instrumento integral que incorpore la multidimensionalidad de la alfabetización en salud^11-12^.

Los cuidadores de niños que requieren cuidados especiales de salud enfrentan desafíos diarios para garantizar una atención segura y apropiada, lo que requiere habilidades para comprender y aplicar la información de salud. Sin embargo, las barreras relacionadas con la alfabetización en salud, como las dificultades para obtener e interpretar información médica, pueden comprometer la toma de decisiones, la adherencia al tratamiento y la seguridad infantil^4^.

Así, identificar las condiciones de alfabetización en salud de una población proporciona información útil y necesaria a las organizaciones y profesionales de salud para mejorar las acciones y servicios de salud^13^. Estudios internacionales aportan cada vez más evidencia sobre la relación entre la alfabetización en salud inadecuada de los cuidadores, la incapacidad de gestionar el cuidado domiciliario y el empeoramiento de los resultados de salud de estos niños, lo que se relaciona con una baja adherencia al tratamiento, errores en la administración de medicamentos en el hogar y un mayor número de hospitalizaciones no programadas^14-17^. Sin embargo, en Brasil, este escenario es poco conocido y no se advierten investigaciones que demuestren las condiciones de alfabetización en salud de esta población, la que justifica la necesidad de este estudio.

Este estudio pretende llenar este vacío identificando las limitaciones y las potencialidades de los cuidadores para navegar por el sistema de salud, obtener y aplicar información y tomar decisiones para el cuidado de estos niños. Para ello, el estudio tuvo como objetivo explorar la alfabetización en salud de cuidadores de niños que requieren cuidados especiales de salud, identificando limitaciones y potencialidades y la relación con variables socioeconómicas, fuentes de información y soporte social.

## Método

### Diseño del estudio

Estudio cuantitativo, transversal, descriptivo y analítico^18^, reportado de acuerdo con las directrices del *Strengthening the Reporting of Observational Studies in Epidemiology* (STROBE)^19^.

### Lugar y periodo

El estudio se realizó entre diciembre de 2022 y abril de 2023 en Porto Alegre-RS, Brasil, y se desarrolló en las unidades pediátricas de un hospital de alta complejidad. El servicio de pediatría cuenta con 131 camas y es referencia nacional para la atención de niños con enfermedades crónicas de mediana y alta complejidad, como enfermedades gastrointestinales, genéticas, oncológicas, hematológicas, neurológicas, enfermedades hepáticas, candidatos a trasplante y trasplantados hepáticos, trasplante de células madre hematopoyéticas, entre otras enfermedades crónicas.

### Población

Cuidadores primarios de niños que requieren cuidados especiales de salud, hospitalizados en unidades pediátricas.

### Criterios de selección y definición de la muestra

Se entiende por cuidador principal o primario a una persona que se identifica como única responsable del niño y de brindarle cuidado diario de manera permanente o parcial, sin remuneración, y sin hallarse necesariamente ligado al niño por vínculos de sangre, sino afectivos. Se consideró niño al individuo que tenía menos de 12 años durante el período de recolección de datos^20^. Para definir la elegibilidad como NANEAS, se estableció que deberían estar presentes en al menos un dominio indicado a través de la aplicación de la versión brasileña del instrumento *Children with Special Care Screener* (CSHCN *Screener*®) entre los tres existentes, a saber, uso de medicamentos, uso de servicios de salud y limitaciones funcionales^21^.

En un principio se realizaron consultas a las historias clínicas electrónicas de los pacientes hospitalizados en unidades pediátricas para identificar a aquellos menores de 12 años. Posteriormente, con el objetivo de rastrear los NANEAS, se aplicó al cuidador principal la versión brasileña del instrumento CSHCN *Screener* ^®(21)^. A los cuidadores principales cuyos niños fueron identificados como NANEAS se les entregaron al mismo tiempo los instrumentos de caracterización de cuidadores y el cuestionario de evaluación de alfabetización en salud: HLQ-Br.

Se incluyó en el estudio a personas mayores de 18 años, que hablaban portugués brasileño (idioma de las versiones validadas de los instrumentos) como primera lengua habitual y que se identificaron como cuidadores principales, en hospitalización y en domicilio, de NANEAS menores de 12 años, que ya habían recibido al menos un alta hospitalaria. No se establecieron criterios de exclusión para la selección de la muestra.

El número mínimo de participantes calculado para la muestra fue de 124 cuidadores, considerando un incremento del 10% por posibles pérdidas y negativas, habiéndose obtenido un total de 138 participantes. El cálculo consideró una potencia del 80%, un nivel de significancia del 5% y un nivel de Cohen de 0,51^22^. Este cálculo se realizó utilizando la herramienta *PSSHealth*, versión *online* ^23^.

### Instrumentos de recolección de datos y variables de estudio

Para identificar a los NANEAS hospitalizados, se aplicó la versión brasileña del CSHCN *Screener*®. Se trata de un instrumento de cribado, desarrollado originalmente en inglés, traducido y adaptado al portugués brasileño, que mostró buena consistencia interna. El instrumento puede identificar y evaluar las demandas de atención de los niños que requieren cuidados especiales de salud relacionados con una condición crónica, más allá de la presencia o no de un diagnóstico clínico^6,21^. El uso de este instrumento se justificó por su sensibilidad para identificar las múltiples dimensiones de las necesidades de salud de los niños. Esto incluye el criterio de mayor riesgo de enfermedades crónicas según la definición original del *Children with Special Care Needs*, que considera condiciones con duración mínima de 12 meses. Aunque los niños de la muestra ya presentaban condiciones crónicas establecidas, el instrumento se aplicó para confirmar la elegibilidad en el momento de la recolección y para identificar los dominios de las necesidades especiales de atención de salud, dado que se trató de un estudio destinado a evaluar la alfabetización en salud de los cuidadores de niños ya identificados con necesidades de cuidados especiales de salud.

El CSHCN *Screener*® contiene cinco elementos y cada uno tiene dos subelementos (a y b), excepto el elemento cinco, que solo tiene uno. Todas las opciones de respuesta son “sí” o “no”. Los subítems se interrogan a partir de una respuesta afirmativa en el respectivo ítem y buscan relaciones entre estas afirmaciones y la existencia de algún problema de salud cuya duración real o esperada sea de al menos doce meses. Cualquiera de los ítems que reciba una respuesta afirmativa, junto con sus respectivos subítems, ya indica que el niño tiene alguna necesidad especial de atención sanitaria. Los cinco ítems se distribuyen en tres dominios de necesidades especiales de atención de salud que requieren atención especializada por parte de los servicios de salud y educativos, a saber, el uso de medicamentos, el uso de servicios de salud y la limitación funcional^21^.

Se aplicó el Cuestionario de Caracterización de NANEAS y cuidadores primarios, desarrollado para este estudio, considerando la relevancia de conocer los factores socioeconómicos, demográficos y culturales de la familia y el historial de salud del niño para un diagnóstico preciso de la alfabetización en salud del cuidador. El cuestionario contiene variables sobre: caracterización de NANEAS - origen de las necesidades de atención sanitaria especial (congénitas, causa adquirida y diagnóstico médico), categorías de demandas de atención (de desarrollo, tecnológicas, medicamentosas, habituales modificadas, mixtas y clínicamente complejas), motivo de la hospitalización actual, fecha de hospitalización y nacimiento, sexo, motivo y fecha de rehospitalización tras el primer alta hospitalaria, número de visitas a urgencias o ingresos hospitalarios en el último año y motivo, utilización de otros servicios de salud; y la caracterización socioeconómica del cuidador ― grado de parentesco con el NANEAS, fecha de nacimiento, sexo, educación, profesión/ocupación, ingreso familiar, si tiene o no otros hijos, existencia de otras personas que requieran de atención especial de salud y dependan de su cuidado, principales fuentes de información de salud (televisión, periódico, radio, internet y otras), ciudad y estado de residencia y existencia de otro cuidador principal.

Para evaluar las condiciones de alfabetización en salud de los cuidadores primarios, se aplicó la versión brasileña del *Health Literacy Questionnaire* (HLQ-Br). El *Health Literacy Questionnaire* es un instrumento multidimensional que fue traducido al portugués brasileño y validado con 794 usuarios adultos del Sistema Único de Salud (SUS) de tres regiones diferentes de Brasil. En este proceso de validación obtuvo buenas propiedades psicométricas. Es un instrumento multidimensional, con 44 ítems distribuidos en nueve escalas que se dividen en dos partes. La Parte 1 tiene cuatro opciones de respuesta entre “totalmente en desacuerdo” y “totalmente de acuerdo” (puntajes del 1 al 4), la Parte 2 tiene cinco opciones de respuesta entre “no puedo hacerlo o siempre es difícil” y “siempre fácil” (puntajes del 1 al 5). Este instrumento no proporciona una puntuación global, sino que puntúa con el promedio individual de las nueve escalas. La puntuación más cercana al límite superior o inferior indica, respectivamente, las potencialidades y las limitaciones de los cuidadores en el cuidado de su salud^24^.

Para aplicar la versión brasileña del HLQ en este estudio, se obtuvo autorización de uso de la Universidad Deakin, Australia, a través de la dirección de correo electrónico “HLQ-info@swin.edu.au”.

### Recopilación de datos

La recolección de datos se realizó en la historia clínica electrónica del paciente y mediante entrevista al cuidador principal de NANEAS, realizada en una sala reservada de las propias unidades pediátricas, garantizando la privacidad. El equipo de investigadores fue previamente capacitado para recolectar datos de historias clínicas y aplicar cuestionarios.

### Procesamiento y análisis de datos

Los datos fueron analizados mediante el programa estadístico *Statistical Package for the Social Sciences* (SPSS/PASW), versión 27.0. En el análisis descriptivo de las variables cuantitativas se utilizó media y desviación estándar, y para el análisis de las variables categóricas se utilizaron frecuencias absolutas y relativas. La decisión sobre el tipo de distribución se basó en el tamaño de la desviación estándar (que no puede exceder la mitad de la media para considerar la distribución simétrica) y la proximidad entre la media y la mediana (diferencia menor al 20%). El nivel de significancia adoptado fue del 5% (p≤0,05).

Para comparar medias se aplicó la prueba t de Student o Análisis de Varianza (ANOVA) junto con la prueba de Tukey. Además, para evaluar la asociación entre variables categóricas se aplicó la prueba Chi-cuadrado de Pearson o la prueba exacta de Fisher. Para evaluar la asociación entre las variables ordinales y las puntuaciones del HLQ-Br, se utilizó la prueba de correlación de Spearman.

### Aspectos éticos

Este estudio tuvo su proyecto aprobado por el Comité de Ética en Investigación de la institución en estudio bajo dictamen N.º 5.751.478. Los aspectos éticos se respetaron y siguieron de acuerdo con la legislación vigente. Los participantes firmaron el Formulario de Consentimiento Libre e Informado.

## Resultados

La muestra estuvo compuesta por 139 cuidadores primarios de NANEAS hospitalizados. La mayoría de estos cuidadores eran del sexo femenino (89,9%), con edad entre 31 y 60 años (62,6%), habían finalizado sus estudios secundarios (55%), eran amas de casa (79%), con renta familiar que oscilaba entre uno y dos salarios mínimos (73,4%) y provenían de diferentes municipios de la región sur de Brasil (95,6%).

De la muestra, 39 cuidadores (28,1%) tenían otra persona dependiente a su cuidado que requería atención sanitaria especial, además del NANEAS. Los cuidadores principales fueron predominantemente madres (84,2%) con otros hijos además del NANEAS (69,1%), y 86 (61,9%) cuidadores principales reportaron contar con el apoyo de un segundo cuidador principal para la atención del niño: el padre en el 38,1% de los casos.

Los participantes eran cuidadores primarios de niños que requerían atención especial de salud relacionada con el uso de medicamentos para una condición crónica (94,2%), utilizaban más los servicios de salud que la mayoría de los niños de la misma edad (100%) y tenían alguna limitación funcional o incapacidad para realizar actividades comunes a la mayoría de los niños (53,2%). De estos niños, 137 (97,8%) tenían más de una necesidad de atención especial de salud y 98 (70,5%) también eran niños dependientes de tecnologías que utilizaban al menos un dispositivo médico.

En el análisis de grupos de combinaciones de dominios de necesidades de atención especial a la salud de NANEAS, sólo tres (2,2%) necesitaron únicamente Servicios de Salud (SS); cinco (3,6%) utilizaron los Servicios de Salud (SS) y presentaron Limitaciones Funcionales (LF), mientras que 62 (44,6%) utilizaron Medicación y SS y la mayoría (69 – 49,6%) de los NANEAS presentaron LF y requirieron el Uso de Medicación y SS.

Como se describe en la Tabla 1, considerando las puntuaciones medias de las nueve escalas del cuestionario HLQ-Br, se identificaron limitaciones en la alfabetización en salud de los cuidadores relacionadas con la Atención activa de salud (Escala 3 - Parte 1), Soporte social para la salud (Escala 4 - Parte 1) y Capacidad para encontrar buena información sobre salud (Escala 8 - Parte 2). Se identificaron potencialidades en la alfabetización en salud de los cuidadores con respecto a la Evaluación de la información sobre salud (Escala 5 – Parte 1) y Capacidad para interactuar activamente con los profesionales de la salud (Escala 6 – Parte 2).

**Tabla 1-:** Puntuaciones medias de las escalas e ítems del HLQ-Br* que indican mayores limitaciones y potencialidades de la alfabetización en salud de los cuidadores primarios de NANEAS hospitalizados^†^(n = 139). Porto Alegre, RS, Brasil, 2023

**Escalas y preguntas del HLQ-Br** *	**Media**	**Desviación estándar**
**Parte 1 (Puntuaciones 1-4)**		
**Escala 1 – Comprensión y apoyo de los profesionales sanitarios**	3,02	0,53
P ^‡^ 17 – Cuento con los profesionales de la salud que necesito para…	3,25	0,71
P ^‡^ 8 – Tengo al menos un profesional de la salud con quien…	2,79	0,91
**Escala 2 – Información suficiente para cuidar la salud**	3,03	0,47
P ^‡^ 23 – Tengo toda la información que necesito para…	3,68	0,64
P ^‡^ 14 – Estoy seguro de tener toda la información que necesito…	3,01	0,73
**Escala 3 – Atención sanitaria activa**	2,82	0,51
P ^‡^ 6 – Paso mucho tiempo involucrado con…	3,40	0,95
P ^‡^ 21 - Hay cosas que hago regularmente para…	2,51	0,70
**Escala 4 – Soporte social a la salud**	2,96	0,59
P ^‡^ 19 – Tengo un fuerte apoyo…	3,24	0,76
P ^‡^ 5 – Cuando me siento enfermo, las personas que me rodean…	2,75	0,78
**Escala 5 – Evaluación de la información de salud**	3,13	0,45
P ^‡^ 7 – Cuando veo nuevas informaciones sobre salud…	3,37	0,62
P ^‡^ 4 – Comparo la información de salud obtenida…	2,94	0,76
**Parte 2 (Puntuaciones 1 – 5)**		
**Escala 6 – Capacidad para interactuar activamente con profesionales de la salud**	4,00	0,57
P ^‡^ 20 – Hacer preguntas a los profesionales de la salud para…	4,39	0,83
P ^‡^ 2 – Asegurarse de que los profesionales sanitarios…	3,60	0,96
**Escala 7 – Navegar por el sistema de salud**	3,70	0,74
P ^‡^ 13 – Asegurarse de encontrar el lugar adecuado para…	4,01	1,07
P ^‡^ 1 – Encontrar el servicio de salud…	3,31	1,08
**Escala 8 – Capacidad para encontrar buena información sobre salud**	3,60	0,64
P ^‡^ 10 – Obtener información sobre salud para…	3,81	0,91
P ^‡^ 18 – Obtener información sobre…	3,37	1,19
**Escala 9 – Comprender la información de salud y saber qué hacer**	3,99	0,57
P ^‡^ 21 – Entender que los profesionales de la salud…	4,38	0,71
P ^‡^ 12 – Leer y comprender la información…	3,81	1,06

*HLQ-Br = Versión brasileña del *Health Literacy Questionnaire* (traducción libre al español); ^†^NANEAS = Niños con Necesidades Especiales de Atención en Salud; ^‡^P = Pregunta

Todos los cuidadores participantes habían transitado al menos una hospitalización con el NANEAS durante los12 meses anteriores a la recopilación de datos para este estudio. De éstos, 30 (21,6%) fueron hospitalizados sólo una vez, 74 (53,2%) fueron hospitalizados de dos a seis veces, 23 (16,5%) fueron hospitalizados de siete a once veces y 12 (8,7%) fueron hospitalizados doce o más veces durante el período.

En la Tabla 2 se describen los coeficientes de correlación entre las puntuaciones medias de las nueve escalas del cuestionario HLQ-Br y las variables medias de número de hospitalizaciones de los NANEAS, nivel de escolaridad e ingreso familiar de los cuidadores primarios. Las asociaciones positivas y estadísticamente significativas (p<0,05) indican la relación entre el nivel de educación, las condiciones de ingreso familiar de los cuidadores y sus limitaciones y potencialidades en alfabetización en salud, como también en relación al número de hospitalizaciones requeridas por el NANEAS.

**Tabla 2-:** Correlación entre las puntuaciones medias de las escalas HLQ-Br* con las características socioeconómicas de los cuidadores primarios de NANEAS^†^y el número de hospitalizaciones de estos niños (n = 139). Porto Alegre, RS, Brasil, 2023

**Escalas HLQ-Br** *	**Nivel de educación**	**Ingresos familiares**	**Hospitalizacion del NANEAS** ^†^
**Parte 1**			
**1 -** Comprensión y apoyo de los profesionales de la salud	r _s_^‡^ =0,23 ^§^	r _s_^‡^ =0,23 ^§^	r _s_^‡^ =0,16
	(p=0,006)	(p=0,006)	(p=0,057)
**2 -** Información suficiente para cuidar de la salud	r _s_^‡^ =0,15	r _s_^‡^ =0,09	r _s_^‡^ =0,17
	(p=0,084)	(p=0,302)	(p=0,053)
**3 -** Atención sanitaria activa	r _s_^‡^ =0,10	r _s_^‡^ =0,09	r _s_^‡^ =0,08
	(p=0,260)	(p=0,292)	(p=0,334)
**4 –** Soporte social para la salud	r _s_^‡^ =0,33 ^§^	r _s_^‡^ =0,32 ^§^	r _s_^‡^ =-0,004
	(p=0,000)	(p=0,000)	(p=0,96)
**5 -** Evaluación de la información sanitaria	r _s_^‡^ =0,17 ^||^	r _s_^‡^ =0,17 ^||^	r _s_^‡^ = 0,19 ^||^
	(p=0,038)	(p=0,044)	(p=0,019)
**Parte 2**			
**6 -** Capacidad para interactuar activamente con profesionales de la salud.	r _s_^‡^ =0,13	r _s_^‡^ =0,15	r _s_^‡^ =-0,12
	(p=0,115)	(p=0,064)	(p=0,158)
**7 –** Navegar por el sistema de salud	r _s_^‡^ =0,62	r _s_^‡^ =0,20 ^||^	r _s_^‡^ =-0,051
	(p=0,467)	(p=0,016)	(p=0,553)
**8 -** Capacidad para encontrar buena información sobre salud	r _s_^‡^ =0,35 ^§^	r _s_^‡^ =0,26 ^§^	r _s_^‡^ =-0,01
	(p=0,000)	(p=0,002)	(p=0,837)
**9 -** Comprender la información de salud y saber qué hacer	r _s_^‡^ =0,22 ^§^	r _s_^‡^ =0,16 ^||^	r _s_^‡^ =0,01
	(p=0,009)	(p=0,049)	(p=0,838)

*HLQ-Br = Versión brasileña del *Health Literacy Questionnaire* (traducción libre al español); ^†^NANEAS = Niños con Necesidades Especiales de Atención en Salud; ^‡^r_s_ = Coeficiente de correlación de Spearman; ^§^Correlación significativa al nivel 0,01; ^||^Correlación significativa en el nivel 0,05

Respecto a la parte 1 del cuestionario HLQ-Br, se observó asociación positiva y estadísticamente significativa entre las variables nivel de educación e ingresos familiares con las escalas 1 - Comprensión y apoyo de los profesionales de la salud (p=0,006), 4 - Soporte social a la salud (p=0,000) y 5 - Evaluación de la información sanitaria (p=0,038; p=0,044). Además, hubo una asociación positiva y significativa entre la variable hospitalizaciones NANEAS y la escala 5 - Evaluación de la información sanitaria (p=0,019) (Tabla 2).

En la Parte 2, se identificó una asociación positiva y estadísticamente significativa entre las variables nivel de educación e ingreso familiar con las escalas 8 - Capacidad para encontrar buena información sobre salud (p=0,000: p=0,002) y 9 - Comprender la información de salud y saber qué hacer (p=0,009; p=0,049). Además, se encontró asociación entre la variable ingreso familiar y la escala 7 - Navegar por el sistema de salud (p=0,016) (Tabla 2).

La mayoría de los cuidadores informaron tener Internet (87%) como una de sus principales fuentes de información de salud, mientras que los profesionales de la salud fueron mencionados como la fuente principal por 19 cuidadores (13,6%) y tres (2,1%) indicaron a otros cuidadores principales como una de sus principales fuentes de información sobre salud.

Se identificaron potencialidades en alfabetización en salud respecto a “Comprensión y apoyo de los profesionales de la salud (Escala 1)” en aquellos cuidadores que reportaron tener a los profesionales de la salud como una de sus principales fuentes de información sobre salud (p=0,002). Respecto a la “Evaluación de la información sanitaria (Escala 5)”, se observaron potencialidades en la alfabetización en salud de aquellos cuidadores que señalaron a internet (p=0,026) como una de las principales fuentes de información sobre salud (Tabla 3).

Se identificaron limitaciones en la alfabetización en salud respecto a “Soporte social a la salud (Escala 4)” y “Evaluación de la información en salud (Escala 5)” en aquellos cuidadores que refirieron no contar con el apoyo de un segundo cuidador principal para el NANEAS (p = 0,039 – p = 0,037), según la comparación de medias descrita en la Tabla 3.

**Tabla 3-:** Asociación de las condiciones de alfabetización en salud de los cuidadores primarios de NANEAS* según el cuestionario HLQ-Br^†^y las variables red de apoyo para el cuidado y fuentes de información en salud (n = 139). Porto Alegre, RS, Brasil, 2023

**Escalas HLQ-Br** ^†^	**E** ^‡^ **1**	**E** ^‡^ **2**	**E** ^‡^ **3**	**E** ^‡^ **4**	**E** ^‡^ **5**	**E** ^‡^ **6**	**E** ^‡^ **7**	**E** ^‡^ **8**	**E** ^‡^ **9**
**Variables**	**M** ^§^ ±DE ^||^	**M** ^§^ ±DE ^||^	**M** ^§^ ±DE ^||^	**M** ^§^ ±DE ^||^	**M** ^§^ ±DE ^||^	**M** ^§^ ±DE ^||^	**M** ^§^ ±DE ^||^	**M** ^§^ ±DE ^||^	**M** ^§^ ±DE ^||^
**FI** ^¶^ - **Otro cuidador**									
No	2,97±0,51	3,01±0,52	2,73±0,53	2,83±0,58	3,03±0,43	4,04±0,54	3,74±0,76	3,58±0,59	4,04±0,50
Sí	3,06±0,55	3,04±0,44	2,88±0,49	3,05±0,59	3,10±0,45	3,97±0,59	3,68±0,73	3,60±0,67	3,97±0,60
Valor p**	0,366	0,750	0,086	0,039	0,037	0,448	0,618	0,855	0,489
**FI** ^¶^ **- *Internet***									
No	2,87±0,44	3,06±0,49	2,93±0,49	2,97±0,56	2,91±0,51	4,00±0,61	3,96±0,93	3,55±0,74	4,10±0,54
Sí	3,05±0,54	3,02±0,47	2,81±0,51	2,96±0,59	3,16±0,43	4,00±0,57	3,66±0,70	3,60±0,62	3,98±0,57
Valor p**	0,192	0,711	0,349	0,925	0,026	0,99	0,115	0,74	0,42
**FI** ^¶^ **- Periódico/Libro**									
No	3,02±0,52	3,02±0,46	2,83±0,50	2,97±0,58	3,10±0,44	4,00±0,58	3,71±0,74	3,57±0,64	3,99±0,56
Sí	3,04±0,62	3,04±0,56	2,780,67	2,83±0,62	3,40±0,47	4,00±0,56	3,63±0,77	3,90±0,47	4,03±0,66
Valor p**	0,915	0,914	0,760	0,452	0,041	0,993	0,751	0,098	0,820
**FI** ^¶^ **- TV/Radio**									
No	3,06±0,53	3,04±0,46	2,83±0,49	2,97±0,59	2,94±0,59	3,15±0,42	3,74±0,69	3,60±0,61	4,03±0,50
Sí	2,91±0,523	2,98±0,49	2,80±0,56	2,94±0,59	3,07±0,52	3,80±0,56	3,60±0,85	3,59±0,72	3,88±0,71
Valor p**	0,14	0,511	0,707	0,815	0,333	0,013	0,335	0,940	0,165
**FI** ^¶^ **- Profesionales de la salud**									
No	2,97±0,52	3,01±0,49	2,85±0,50	2,98±0,58	3,11±0,45	4,00±0,58	3,72±0,75	3,61±0,65	4,01±0,58
Sí	3,38±0,49	3,13±0,30	2,66±0,52	2,87±0,62	3,24±0,46	3,95±0,55	3,57±0,63	3,50±0,55	3,90±0,48
Valor p**	**0,002**	0,320	0,134	0,468	0,458	0,725	0,397	0,484	0,446
**FI** ^¶^ **– Cuidadores**									
No	3,02±0,53	3,03±0,47	2,84±0,50	2,97±0,59	3,12±0.45	4,00±0,58	3,71±0,74	3,60±0.64	3,99±0,57
Sí	3,25±0,47	2,91±0,62	2,13±0,46	2,60±0,34	3,40±0,52	4,06±0,41	3,22±0,63	3,53±0,46	4,00±0,52
Valor p**	0,47	0,676	0,017	0,280	0,304	0,844	0,257	0,853	0,997

*NANEAS = Niños con Necesidades Especiales de Atención en Salud; ^†^HLQ-Br = *Health Literacy Questionnaire*; ^‡^E = Escala; ^§^M = Media; ^||^DE = Desviación estándar; ^¶^FI = Fuente de información de salud; **valor p = Prueba t de Student

## Discusión

El estudio innova al analizar las condiciones de alfabetización en salud de los cuidadores de NANEAS, utilizando un instrumento de evaluación multidimensional. Esto se traduce en el reconocimiento de las limitaciones y potencialidades respecto a las habilidades funcionales, interactivas y críticas de estos cuidadores, asociándolas a sus condiciones socioeconómicas y a las demandas de cuidado que requieren los NANEAS^2^.

Los NANEAS requieren una alta demanda de cuidados específicos y complejos, además requieren de un tratamiento multidisciplinario y seguimiento durante un largo periodo. El manejo de este complejo escenario requiere conocimientos específicos, habilidades técnicas y de comunicación por parte del cuidador principal^25-26^.

En este estudio, todos los NANEAS fueron identificados como dependientes de los servicios de salud y tuvieron un número significativo de hospitalizaciones durante un período de un año. La mayoría de los NANEAS fueron reconocidos en la combinación de los tres dominios de necesidades especiales de salud, además de depender de dispositivos tecnológicos para mantener la vida. Estos hallazgos ponen de relieve la complejidad de la atención que requieren estos niños y que muchas veces es requerida por cuidadores en el hogar, sin que exista preparación para el alta ni soporte adecuado de la red de servicios de salud^27-29^.

El cuidador principal es reconocido como parte fundamental de la atención segura y de calidad del NANEAS en el hogar. Desempeña un papel crucial, junto con los profesionales sanitarios, en la toma de decisiones relativas a las necesidades de salud de este niño y, sobre todo, en la gestión y ejecución de las demandas de atención domiciliaria. Además de los desafíos que genera la complejidad del cuidado, los cuidadores también enfrentan una serie de dificultades impuestas por el propio sistema de salud^(15,30-31)^.

Para afrontar estas demandas y superar las dificultades impuestas, el cuidador necesita desarrollar habilidades cognitivas y sociales que le permitan navegar por el sistema de salud, obtener, comprender y aplicar la información necesaria para gestionar el cuidado, emitir juicios y tomar decisiones sobre las necesidades y particularidades de estos niños^26,32^.

Los resultados de este estudio demostraron condiciones razonables de alfabetización en salud entre los cuidadores, reconocidas en las escalas del cuestionario aplicado, aunque también se identificaron limitaciones relacionadas con las habilidades funcionales, interactivas y críticas^2^. Cabe señalar que la alfabetización en salud de un individuo no es estática. Factores situacionales como un entorno desconocido, una interacción intimidante y factores emocionales pueden afectar temporal y negativamente las condiciones de alfabetización en salud y la capacidad de estos cuidadores para gestionar la atención que necesitan los NANEAS^32-34^.

Se advierten limitaciones en cuanto a la atención sanitaria activa por parte de los cuidadores en este estudio con respecto a las habilidades interactivas y críticas. Estos cuidadores, a pesar de tener las habilidades y conocimientos necesarios para practicar el autocuidado, no actúan para promoverlo, ya que no lo ven como una prioridad. Hallazgos similares se identifican en otros estudios en los que el cuidador principal se dedica exclusivamente a la demanda intensiva de los cuidados complejos requeridos por los NANEAS y termina por no involucrarse en su propio cuidado, lo que muchas veces resulta en una sobrecarga física, emocional y social^(31,35-37)^.

Como era de esperar, las madres se identificaron mayoritariamente como las principales cuidadoras de los NANEAS durante la hospitalización y en el hogar, como se ha identificado globalmente en otros estudios^(25-26,~34,38)^. Los determinantes sociales que mostraron una relación significativa con el estado de alfabetización en salud de estos cuidadores fueron el nivel de educación y el ingreso familiar, como se ha confirmado en otros estudios^(17,39-41)^.

La falta de apoyo social también expone a los cuidadores a una sobrecarga física y mental, lo que puede afectar su salud, bienestar y calidad de vida, además de dificultar la práctica de habilidades de alfabetización en salud previamente adquiridas y alterar la capacidad para realizar cuidados a los NANEAS^33,37^. En este estudio, la mayoría de los cuidadores informaron haber recibido un amplio soporte de su red de apoyo primaria, incluida la asistencia de un segundo cuidador para turnarse en la prestación de cuidados, pero informaron también no sentirse comprendidos por sus seres queridos cuando se sienten enfermos.

En otro estudio, las madres que cuidan a NANEAS reportan un apoyo social limitado, manifiestan no poder contar con el apoyo del padre del niño para brindarle cuidados en casa y sienten un gran agotamiento mental y físico. Respecto a las relaciones sociales familiares, refieren sentimientos de aislamiento, incomprensión, falta de empatía y dificultad para comunicarse. Si bien no reciben un apoyo familiar adecuado, a través del intercambio de experiencias estas cuidadoras experimentan fuertes vínculos con otras madres de NANEAS, por quienes se sienten comprendidas y apoyadas^42^.

Recibir apoyo social de amigos y familiares se ha destacado como esencial para gestionar las necesidades del niño, compartir el cuidado y, sobre todo, para incorporar nueva información sobre los desafíos que se enfrentan ante la complejidad que implica el cuidado de un NANEAS. Condiciones más avanzadas de habilidades críticas interactivas y comunicativas favorecen el fortalecimiento de las redes de apoyo primarias y secundarias y posibilitan una mayor extracción de información de diferentes fuentes^32,37^.

La red social secundaria debilitada también contribuye a las limitaciones en la alfabetización en salud de los cuidadores. Estudios identifican a las instituciones de salud y asistencia social como una red de apoyo secundaria y describen el vínculo entre cuidadores y profesionales de la salud como conflictivo, frágil o quebrado. La fragilidad de este vínculo está asociada a la fragmentación en la organización de los servicios de salud, mala calidad de la atención, limitada responsabilidad por parte de los profesionales de la salud, brechas en la comunicación, orientación insuficiente y distanciamiento de los profesionales con relación al cuidador^37,43^.

En el caso de los cuidadores de NANEAS, la falta de habilidades para buscar información y desenvolverse en el sistema de salud por sí mismos constituye una barrera muy relevante para la atención domiciliaria e indica limitaciones en la alfabetización funcional e interactiva en salud de estos cuidadores^32^. Estos niños requieren el uso frecuente de servicios de salud y profesionales de múltiples especialidades. Cuanto mayor sea el equipo de profesionales, más visitas a los servicios de salud deberá gestionar el cuidador, lo que requiere habilidades avanzadas de comunicación y coordinación^9,38^.

Las dificultades en la navegación suelen estar causadas por el propio sistema sanitario. Estudios que evalúan el itinerario terapéutico de los NANEAS identifican la falta de preparación de los profesionales de la salud y la fragmentación, desarticulación, falta de estructura y precariedad de los servicios de salud como barreras para el acceso y navegación en la red de servicios de salud^(31,44-45)^. La comunicación entre los profesionales de la salud responsables del tratamiento del niño es percibida por los cuidadores como escasa e insuficiente, al igual que la comunicación entre profesionales y cuidadores^37^.

Para satisfacer las demandas de atención que requiere el NANEAS, el cuidador debe tener acceso a una variedad de información específica y a menudo compleja. Dadas las debilidades en la comunicación, preparación y orientación para la transición del cuidado por parte de los profesionales y servicios de salud^46^, la incapacidad de acceder por sí mismo a la información de salud se vuelve aún más preocupante.

En este estudio, la mayoría de los cuidadores reconocieron a Internet como una de las principales fuentes de información de salud y pocos identificaron a los profesionales de la salud en este rol. El reconocimiento de las redes digitales y sociales, así como de los profesionales sanitarios como fuentes de información por parte de los cuidadores de NANEAS, también se ha descripto en otros estudios, en los que se valora la información de los profesionales, pero con pautas poco compatibles y aplicables a la realidad de la familia y del niño. En estos casos se optó por el intercambio de experiencias con otros cuidadores de niños con necesidades iguales o similares^47^. Al igual que en este estudio, otros cuidadores de NANEAS también fueron reconocidos como una fuente importante de información, intercambio de experiencias y alternativas para cualificar el cuidado al NANEAS.

En otro estudio, se encontró que los cuidadores de niños con enfermedades crónicas tienen más probabilidades de acceder a las redes sociales que discuten la salud del niño, a pesar de clasificarlas como las fuentes menos confiables de información de salud en línea. Además, cuando encontraron información que contradecía el consejo del profesional, la mayoría de los padres informaron haber seguido el consejo del profesional o haber consultado con él antes de tomar una decisión^48^.

En esta investigación se identificaron potencialidades en la alfabetización interactiva en salud de los cuidadores en lo que se refiere a la capacidad de interactuar activamente con los profesionales de la salud. Aunque la mayoría no identifica a los profesionales de la salud como una fuente de información sobre salud, se sienten capaces de buscar orientación adicional de estos profesionales, haciendo preguntas para obtener la información que necesitan. Este resultado se atribuyó a la larga experiencia y coexistencia de estos cuidadores con los profesionales y servicios de salud, debido al uso frecuente de los mismos servicios para atender las demandas de atención del NANEAS^37^.

También se reconocieron potencialidades en la alfabetización crítica en salud de los cuidadores respecto a la capacidad de evaluar información de salud, identificándose una asociación estadísticamente significativa entre la escala correspondiente al ítem del cuestionario aplicado con aquellos cuidadores que utilizan internet como fuente de información de salud.

Es importante destacar que *internet* puede ser una herramienta valiosa de comunicación y adquisición de información sobre salud para estos cuidadores, por desarrollar la capacidad de interacción social, mayor conocimiento sobre la salud, y mejorar la autonomía y la autoconfianza para la gestión del cuidado. Al mismo tiempo, también puede convertirse en un gran riesgo para la salud tanto de los cuidadores como de los niños, ya que es una fuente de información a menudo poco fiable e incompleta. Es necesario que el cuidador tenga la capacidad de identificar fuentes de información buenas y confiables y que pueda distinguir la información contradictoria^(46,49-50)^. Ante este escenario, es necesario evaluar la alfabetización digital en salud de esta población.

Este estudio se destaca por dar voz a los cuidadores de NANEAS, reconociendo sus desafíos diarios en la búsqueda de información y la toma de decisiones para asegurar el bienestar de sus niños. Por haber utilizado instrumentos validados internacionalmente, ofrece un retrato fiel de la realidad de estos cuidadores y aporta una perspectiva inédita sobre esta temática en Brasil. Además de contribuir desde el punto de vista científico, refuerza la necesidad de acercar los profesionales de la salud a las familias y robustecer la alfabetización en salud y la autonomía de estos cuidadores.

Las limitaciones de este estudio se relacionan con la recolección de datos realizada en una sola institución de salud, lo que puede no representar la realidad de los cuidadores en otros contextos, como aquellos que acceden a servicios de menor complejidad o que enfrentan diferentes desafíos regionales en el sistema de salud, además de la ausencia de un enfoque cualitativo que explore las causas subyacentes de las condiciones multidimensionales de la alfabetización en salud de los cuidadores. Asimismo, el estudio fue transversal, lo que no permitió el análisis de los cambios en la alfabetización en salud.

Por lo tanto, se sugieren nuevos estudios en más instituciones de salud y con un enfoque mixto y longitudinal, que permitan desarrollar intervenciones amplias y personalizadas para atender las necesidades de alfabetización en salud de los cuidadores de NANEAS.

## Conclusión

El estudio innova al explorar la alfabetización en salud multidimensional de los cuidadores de NANEAS a nivel nacional, utilizando la asociación de instrumentos internacionales sólidos y ampliamente aplicados, tanto para el cribado y clasificación de NANEAS en dominios de necesidades, como para evaluar las condiciones de alfabetización en salud del cuidador primario más allá de una mera capacidad o limitación cognitiva.

Los hallazgos revelan la influencia del nivel de escolaridad del cuidador y del ingreso familiar en las condiciones de alfabetización en salud, además de identificar a *internet* como la principal fuente de información de estos cuidadores. La mayoría de estas personas tienen una capacidad limitada para actuar en favor de su propia salud y bienestar y cuentan con un apoyo social limitado, a pesar de contar con el soporte de un segundo cuidador para cubrir las necesidades del NANEAS. Además, se sienten incomprendidas y sin apoyo por parte de sus allegados cuando se trata de su propio estado de salud. Asimismo, tienen una capacidad limitada para encontrar por sí mismos buena información sobre salud.

Cabe resaltar la importancia de reconocer las limitaciones y el potencialidades de la alfabetización en salud en esta población como un recurso importante que posibilita la implementación de intervenciones educativas asertivas por parte de los servicios y profesionales de salud responsables de promover la alfabetización en salud, fortalecer la atención activa, mejorar el acceso, la comprensión y el seguimiento de las orientaciones y aumentar la calidad y seguridad de la atención.

## Data Availability

Los conjuntos de datos relacionados con este artículo estarán disponibles previa solicitud al autor correspondiente.
